# Comprehensive Analysis of Gene Expression Profiles of the Beet Armyworm *Spodoptera exigua* Larvae Challenged with *Bacillus thuringiensis* Vip3Aa Toxin

**DOI:** 10.1371/journal.pone.0081927

**Published:** 2013-12-02

**Authors:** Yolanda Bel, Agata K. Jakubowska, Juliana Costa, Salvador Herrero, Baltasar Escriche

**Affiliations:** 1 Department of Genetics, University of Valencia, Burjassot, Valencia, Spain; 2 Department of Applied Biology, UNESP, Jaboticabal, Sao Paulo, Brazil; University of Tennessee, United States of America

## Abstract

Host-pathogen interactions result in complex relationship, many aspects of which are not completely understood. Vip proteins, which are *Bacillus thuringensis* (Bt) insecticidal toxins produced during the vegetative stage, are selectively effective against specific insect pests. This new group of Bt proteins represents an interesting alternative to the classical Bt Cry toxins because current data suggests that they do not share the same mode of action. We have designed and developed a genome-wide microarray for the beet armyworm *Spodoptera exigua*, a serious lepidopteran pest of many agricultural crops, and used it to better understand how lepidopteran larvae respond to the treatment with the insecticidal protein Vip3Aa. With this approach, the goal of our study was to evaluate the changes in gene expression levels caused by treatment with sublethal doses of Vip3Aa (causing 99% growth inhibition) at 8 and 24 h after feeding. Results indicated that the toxin provoked a wide transcriptional response, with 19% of the microarray unigenes responding significantly to treatment. The number of up- and down-regulated unigenes was very similar. The number of genes whose expression was regulated at 8 h was similar to the number of genes whose expression was regulated after 24 h of treatment. The up-regulated sequences were enriched for genes involved in innate immune response and in pathogen response such as antimicrobial peptides (AMPs) and *repat* genes. The down-regulated sequences were mainly unigenes with homology to genes involved in metabolism. Genes related to the mode of action of Bt Cry proteins were found, in general, to be slightly overexpressed. The present study is the first genome-wide analysis of the response of lepidopteran insects to Vip3Aa intoxication. An insight into the molecular mechanisms and components related to Vip intoxication will allow designing of more effective management strategies for pest control.

## Introduction

The beet armyworm *Spodoptera exigua* (Hübner; Lepidoptera: Noctuidae) is a highly dispersive, polyphagous species that is a serious pest of cotton, alfalfa, tomatoes, sugar beets, and many other agricultural and flower crops worldwide [1]. The concern in today's society about the ecological damage caused by the abuse of chemical insecticides as well as the ability of insects to develop resistance to them [1] has led more attention on biological insecticides as alternatives for controlling pests. *Bacillus thuringiensis* (Bt) is the most widely used commercial microbial pathogen. Its spores and crystalline insecticidal proteins (Cry proteins) have been used to control insects since 1938 [2]. In 1996, a novel class of insecticidal proteins isolated from Bt and expressed during the vegetative growth phase (hence the name Vegetative Insecticidal Proteins or Vip proteins), was first reported [3]. These toxic Bt proteins represent an interesting complement to Cry toxins because similarly to Cry proteins, they show high and specific activity against a wide range of agriculturally important lepidopteran larvae [4]. Since both Bt toxins (Cry and Vip) provide excellent control of target pests such as *S. exigua* [5,6] with minimal environmental impact, transgenic crops expressing Cry toxin alone, and recently both Cry and Vip toxins, have been developed and are being increasingly used worldwide [7,8]. This combination of Bt proteins in transgenic plants offers the possibility for targeting a wider range of insects and minimizes the risk of resistance outbreaks in the field.

The mode of action of the insecticidal Cry toxins has been extensively studied for more than 20 years. However, some aspects remain unclear. It is commonly accepted that these crystal proteins need to be solubilized in the insect gut to be processed to the active form, which binds to specific receptors in the brush border epithelial midgut cells. This binding leads to cell lysis, and eventually insect death. The molecular mechanism by which this occurs is not completely understood and differs depending on the binding model [2,9,10]. The available information mainly supports the notion that these toxins act by forming pores [11]. Although little is known about the mode of action of Vip proteins like Vip3Aa, it has also been described to act through the formation of pores in the midgut epithelial cells [12,13]. As with Cry proteins, Vip proteins are ingested either as a protoxin or in the processed toxin form [6,14], and produce similar effects in the insect midguts, causing eventually the lysis of midgut cells [15]. However, Vip proteins have different properties than their Cry counterparts in several key steps of their mode of action, including the binding to midgut receptors [4,12,13,15]. 

The insect midgut is where activated Cry and Vip toxins bind to and initiate cytotoxicity. Freitak and coworkers [16] suggested that in addition to being an organ of digestion and resource assimilation, the midgut epithelial tissue is also an immune response-sensing organ, as they observed that non-pathogenic bacterial feeding could trigger an immune response cascade in the Lepidoptera *Trichoplusia ni*.

In insects, immunity consists of the combination of cellular responses (phagocytosis, encapsulation and melanization of invading microorganisms) and humoral responses (e.g. antimicrobial peptides secreted to the hemolymph) [17,18]. Also, the immune response in insects can be classified as either systemic or local, where the specific tissue (e.g. the gut in the instance of oral intoxication processes) responds locally to the damage [19,20]. Insects respond to microbial ligands mainly through the activation of the stress pathways Toll, Imd, JAK/STAT, JNK, and MAPK p38 [19,21]. Eukaryotic non-immune cells (such as epithelial cells) have evolved various defense responses to cope specifically with pore forming toxins, such as through the activation of the MAPK p38 and JNK pathways [22]. In fact, the MAPK p38 pathway is activated to protect the nematode Caenorhabditis elegans or HeLa cells against the Bt Cry5 toxin [22,23,24], and it is activated in Lepidoptera and Diptera after Cry-toxin intoxication [25]. Some studies indicate that *S. exigua* also responds to bacterial infection through the general mechanisms involved in immune response in insects, producing antimicrobial peptides (AMPs) such as cecropins [26], gloverins [27], or attacins [28], as well as producing “REsponse to PAThogen” (REPAT) proteins in the midgut after Cry1Ca intoxication [29]. 

Recent studies have attempted to characterize the defense response of insects to Bt or Cry intoxication by proteomic analysis and transcriptional profiling approaches [30-37]. Moreover, these types of analyses have also been used to gain insight into the mode of action of Bt Cry toxins by comparing the transcriptional profiles of resistant and susceptible insects [38-41]. In this study, microarray technology was employed to characterize the defense response of *S. exigua* to Vip3Aa intoxication by monitoring gene expression levels after treatment with a sublethal dose of the insecticidal protein. A custom microarray containing more than 29,000 unigenes from a S. *exigua* transcriptome [42] was used for comparison of Vip3Aa-treated and non-treated larvae at two different times post-treatment. The results were validated by quantitative qRT-PCR of selected genes that we identified as having different expression patterns. The transcriptional profiling could allow for a better understanding of Vip protein action in the midguts of intoxicated larvae, and could provide clues about the larval midgut response mechanisms associated with oral Vip intoxication, useful information for future biocontrol strategies.

## Materials and Methods

### Insects, bacteria and toxin

*S. exigua* larvae from the FRA colony kindly provided by M. López-Ferber (INRA, St.-Christol les Alés, France) [33], were used in the experiments. The colony was reared at 25°C, with a relative humidity of 70%, and a photoperiod of 16 h:8 h (light: dark), on an artificial diet [43].

The gene encoding the Vip3Aa protein (NCBI accession AAC37036) cloned into the pMaab9 plasmid, was kindly supplied by Bayer BioScience N. V. (Ghent, Belgium). The *Escherichia coli* WK6 strain was used as the expression host strain. For protein production, *E. coli* cultures were induced with 1 mM IPTG. Cells were pelleted by centrifugation at 8,800 *g* at 4°C for 30 min, frozen at -20°C, and subsequently lysed by a 30 min incubation at 37°C in 20 mM phosphate buffer (pH 7.4) containing 0.5 M NaCl, 3 mg/ml lysozyme, 10 µg/ml DNAse, and 0.1 mM PMSF. The lysate was sonicated on ice and centrifuged at 27000 *g* at 4°C for 30 min. The supernatant (containing the Vip3Aa1 protein), was filtered through 45 µm filters and stored at -20°C until use in the feeding experiments. The concentration of Vip3Aa toxin in the supernatant was determined by densitometry after SDS–PAGE electrophoresis, using the 1D Manager Software (TDI, Madrid, Spain). The *E. coli* control strain was cultured and processed in the same manner as performed for the Vip3Aa-producing *E. coli* strain.

### Treatment of *S. exigua* larvae with Vip3Aa

To synchronize the insects, late third instar *S. exigua* larvae (L3) were selected the day before the feeding experiments. The following day, approximately 16 h after the selection, the newly moulted L4 larvae were separated and exposed, individually, to a dose of Vip3Aa of 111 ng/cm^2^, which produced a 99% growth inhibition (Figure S1). As a control, the filtered supernatant obtained from the *E. coli* control culture, diluted to the same degree as was required to dilute the Vip3Aa-containing supernatant, was used to feed the larvae.

Three independent biological replicates of the Vip3Aa feeding experiments were performed. In each, sixteen larvae were exposed to the supplemented food for 8 h and 24 h. After these times, only larvae that had fed (as determined by observing the food bites) were selected for midgut dissection. At least seven larvae were used for each time point. Midguts of the larvae from each treatment (8 h or 24 h) were pooled for further processing.

### Microarray design

A 44K Agilent oligonucleotide chip was designed using the eArray application from Agilent (Agilent Technologies, Wilmington, DE, USA) and included 29,102 unigenes from *S. exigua* (GEO Acc. No. GPL17775). The sequences of *S. exigua* were obtained from an *S. exigua* transcriptome sequencing project, described elsewhere, specifically designed to be enriched in pathogen-induced genes [42]. Most of the unique assembled sequences (unigenes) in the microarray were represented by two 60-mer oligonucleotide probes, designed to target different sections of each unigene. 

### RNA purification, labeling, and hybridization

The RNA from *S. exigua* midguts was purified using RNAzol reagent from Molecular Research Center, Inc. (Cincinnati, OH, USA), and purified using the RNAeasy Kit (Qiagen GmbH, Hilden, Germany) following the protocols provided by the manufacturers. The quality of RNA was assessed with an Agilent 2100 Bioanalyzer using the Eukaryote Total RNA Nano protocol.

Agilent One-Color Spike-in Mix was added to the purified RNA and 600 ng of total RNA was used for complimentary RNA (cRNA) synthesis. From the resulting cRNA, 165 µg were fluorescently labeled with cyanine-3-CTP 1, fragmented, and hybridized to the S. *exigua* microarray chip following the One-Color Microarray-Based Gene Expression Analysis (Quick-Amp labeling) protocol from Agilent, as described in Jakubowska et al. [44]. RNA labeling and hybridization, as well as array scanning and data extraction, were performed by the Microarray Analysis Service of the Principe Felipe Research Centre (CIPF), Valencia, Spain. Microarray results are available at NCBI, GEO Acc. No. GSE51195.

### Microarray data analysis

*S. exigua* microarray chips were scanned using a G2505B Agilent scanner and data were extracted using Agilent Feature Extraction 9.5.1 software. Before data analysis, hybridization quality control reports were verified for being correct. Data analysis was performed using free Babelomics 4.3 software (available online: http://babelomics.bioinfo.cipf.es/) [45]. First, all arrays were normalized using spike-ins and quantile normalization methods. Normalized arrays for the samples treated with Vip3Aa were compared to the normalized arrays for the controls at the two time points (8 h and 24 h after treatment), and expressed as fold-change in the expression. Fold-change is defined here as a difference in log_2_ values between treated and control sample, and later reported as linear ratios. The thresholds of fold-change > 2 and p-value < 0.05 were applied to consider a gene as regulated compared with control. Previous studies showed that fold change values together with a nonstringent statistical p-value cutoff provided increased consistency in the analysis of Gene Ontology terms and pathways affected [46] and generate more reproducible results [47-50]. Therefore, the false discovery rate (FDR) [51] has not been used. The FDR values in this study ranged from 0.002 (24 h of Vip3Aa treatment) to 0.126 (8 h of Vip3Aa treatment). It is worth noting that the numbers of regulated unigenes before applying statistical t-test were very similar to the numbers of unigenes when t statistics was included, which suggested a high repeatability of the biological replicates.

Annotations of the unigenes were performed using Blast2GO [52]. Functional clustering of regulated genes, while maintaining the applied thresholds, was performed using DAVID version 6.7 software [53]. The 1,470 regulated unigenes with homology to *Bombyx mori* genes that were admitted by DAVID, were then analyzed using the *B. mori* gene list as a background for functional enrichment analysis. Resulting clusters were ranked according to the Enrichment Score, which is the overall score for the whole group of terms and is calculated based on the EASE enrichment scores of all members of the group. The EASE enrichment score was calculated using the Fischer Exact test with the p-value threshold set to 0.05.

### Microarray data validation by quantitative real-time polymerase-chain reaction

To confirm the microarray data, 19 regulated genes were validated by quantitative PCR (qRT-PCR). Primers for the analysis (Table S1) were designed using Primer Express software (V 2.0.0, Applied Biosystems, Foster City, CA, USA) and verified *in silico* using the GenosysOligoMail ver. 2.0 program (Genosys, Sigma-Aldrich, TX, USA). The suitability of the primers was further assessed in the qRT-PCR working conditions. The *ATP synthase* subunit *C* house-keeping gene was used as an internal control for normalization of the samples [29,33,44,54]. The cDNA was synthesized from 1 µg of RNA treated with DNase I (Invitrogen, Life Technologies Corporation, CA, USA) by reverse transcription using oligo-d(T) primer and SuperScript II Reverse Transcriptase (Invitrogen) according to manufacturer protocols. The qRT-PCRs were carried out in an ABI PRISM® 7000 Sequence Detection System (Applied Biosystems). Reactions were performed using Power SYBR Green PCR Master Mix (Applied Biosystems) with 5 µl cDNA template (12.5 ng) in a final reaction volume of 25 µl. Forward and reverse primers were added to a final concentration of 300 nM. Expression ratios were calculated using the formula ΔΔCt = (Ct_gene of interest, treated_ – Ct_reference gene, treated_)-(Ct_gene of interest, control_ – Ct_reference gene, control_). The final and absolute gene regulation values (or fold-change values) were obtained as 2^-ΔΔCt^, and were expressed as 2^-ΔΔCt^ for up-regulated unigenes and as 1/(2^-ΔΔCt^) for down-regulated unigenes, thus allowing a better understanding of down-regulation intensities.

## Results and Discussion

### Microarray data analysis

The transcript profiles of Vip3Aa-treated larvae as compared to control larvae were assessed using a custom *S. exigua* microarray containing 38,174 probes representing more than 29,000 *S. exigua* unigenes. Microarray probes were designed based on the unigenes from a S. *exigua* transcriptome designed to be enriched for pathogen-related genes [42]. A sublethal concentration of Vip3Aa protein (causing 99% growth inhibition) was used to elicit changes in gene expression after 8 and 24 h of treatment.

The data obtained showed that 5526 unigenes were transcriptionally regulated, representing 19% of all unigenes present in the array. This is high when compared with other analyses of transcriptional changes after microbial infection or Cry intoxication in lepidopterans (from 1 to 11%) [55-57], coleopterans (about 1%) [34], or dipterans (around 7%) [20]. This wide transcriptional change suggests a strong response of the organism to Vip intoxication, even taking into account the characteristics of the transcriptome represented in the microarray, aimed to be enriched in pathogen-induced genes [42].

**Figure 1:**
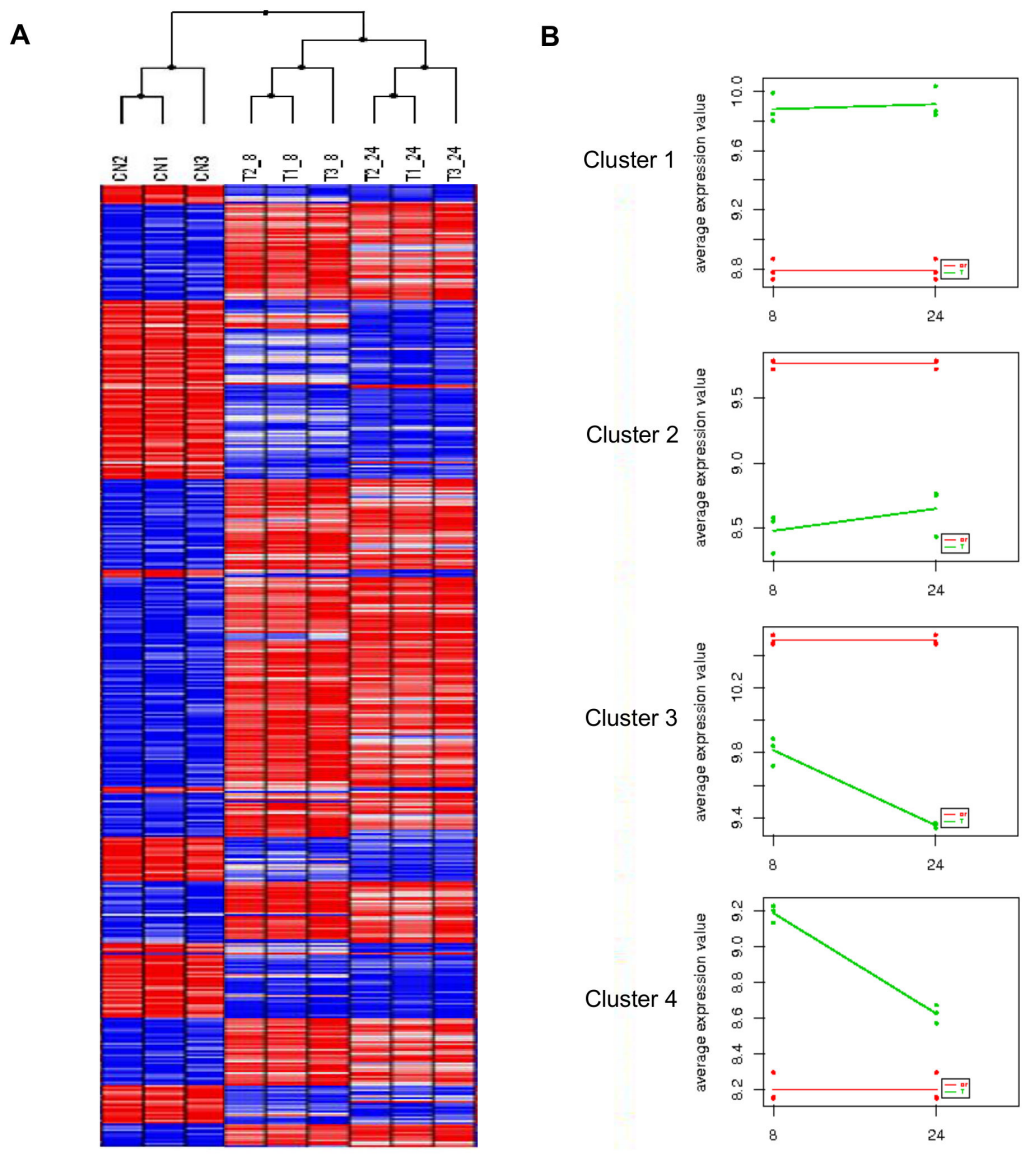
Differentially regulated genes during Vip3Aa intoxication. (A) Clustered heat map of regulated unigenes generated by Babelomics software. (B) Clustered temporal expression profiles of the regulated unigenes over time, generated by Babelomics software. Figure only shows clusters that included more than 500 members. Red: control unigenes. Green: regulated unigenes.

The heat map generated from the microarray data (Figure 1A) showed that the biological replicates grouped together, indicating the robustness of the results. In addition, the genes regulated at 8 and at 24 h grouped together and were separate from the controls. Analysis of the expression profiles over time, grouped the regulated genes into nine clusters. The clusters that included more than 500 genes are represented in Figure 1B and indicate that most of the regulated unigenes showed the same type of regulation at both time points, either up-regulated (3,157 and 589 unigenes, Clusters 1 and 4 respectively) or down-regulated (1,715 and 1,304 unigenes, Clusters 2 and 3, respectively).The full list of unigenes belonging to each Cluster is provided in Table S2.

**Figure 2:**
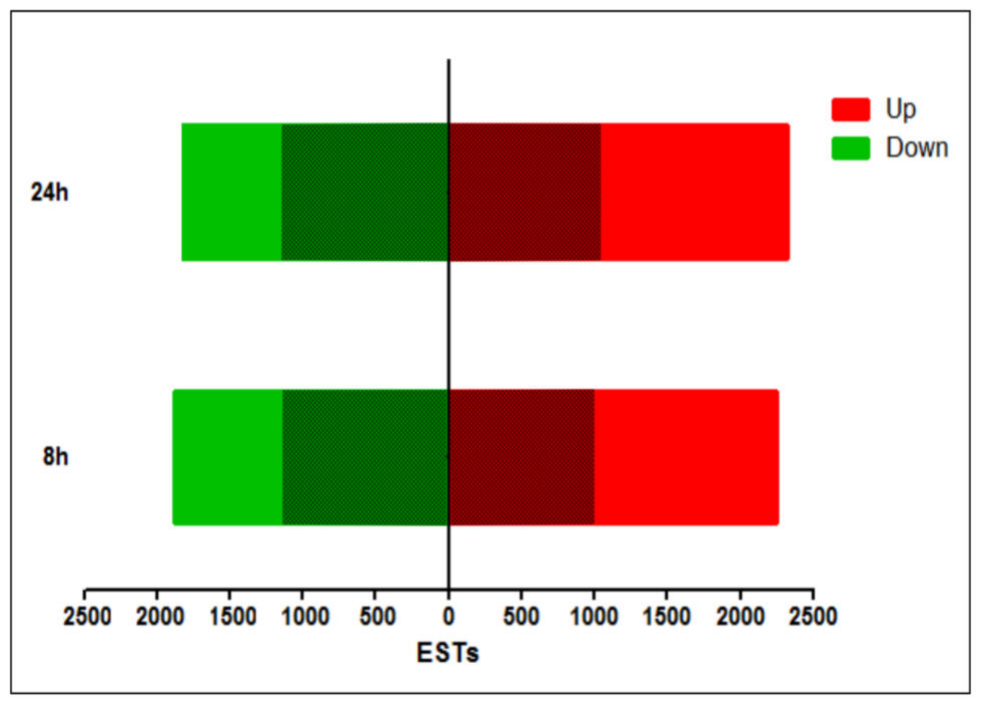
Gene expression overview of regulated unigenes in *S. exigua* midguts. The number of genes up- and down-regulated after 8 h and 24 h of Vip3Aa feeding is shown. Shaded areas indicate the proportion of unigenes with homologies in databases.

Of the 5,526 regulated unigenes, there were almost the same number of regulated genes after 8 h than after 24 h of intoxication (4,121 *vs.* 4,123, respectively; Figure 2). An overview of previous reports on time course transcriptional responses after intoxication with insecticidal proteins or bacterial feeding, shows that the response depends on the insect and on the toxic agent [30,31,34,55-58]. When the up- and down-regulated unigenes were considered separately, the number of regulated genes that resulted was similar at both time points (2,243 and 2,323 up, and 1,878 and 1,800 down, after 8 h and 24 h respectively; Figure 2). These results are in contrast with those described in experiments of intoxication with Bt Cry toxins where the proportion of down-regulated genes was greater than that of up-regulated genes [30,34]. However, our results resembled the ones observed in insects fed with whole bacteria or virus [20,56,57]. It should be noted that the levels of down-regulation achieved by the most repressed genes (around 660-fold) were greater than the levels of overexpression achieved by the most up-regulated genes (around 160-fold).

**Figure 3:**
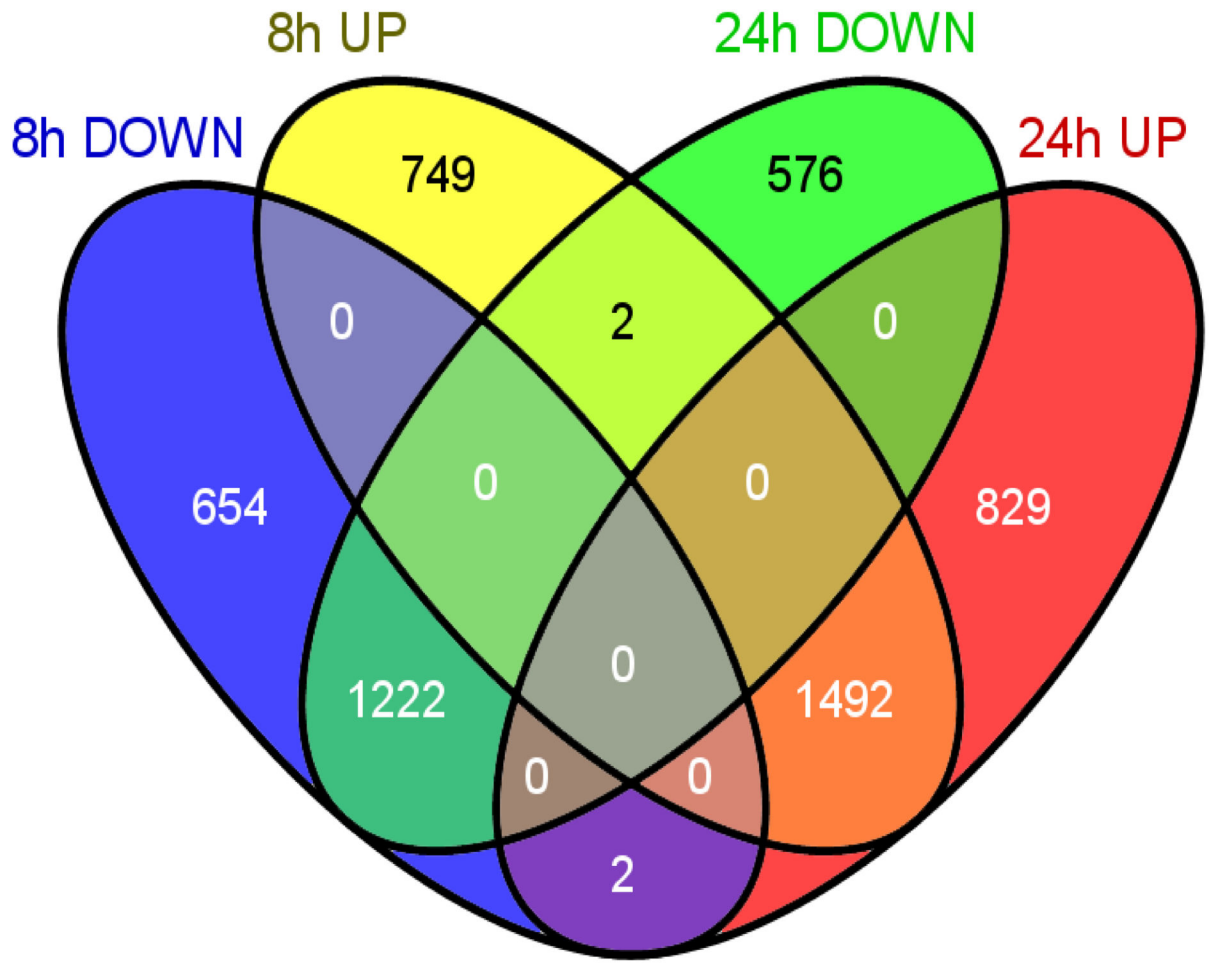
Venn diagram showing up- and down-regulated unigenes at 8 h and 24 h of Vip3Aa feeding.

The distribution of up- and down-regulated unigenes according to length of treatment is summarized in Figure 3. About half of all regulated unigenes exhibited altered expression levels at both 8 h and 24 h of Vip3Aa treatment (1,222 and 1,492, that together account for 49.2% of all regulated unigenes), whereas about a quarter of genes responded only at 8 h (749 up and 654 down, that together account for 25.4% of the regulated genes), and another quarter responded only after 24 h (829 up and, 576 down, a 25.4% of regulated genes).

### Global effect of Vip3Aa treatment on *S. exigua* midgut

Around 40% of the up-regulated and about 60% of the down-regulated unigenes showed homology to known genes from public sequence databases as assessed by Blast2GO annotation (Figure 2). The list of unigenes with homologies in databases, regulated at both 8 h and 24 h, or regulated only at 8 h or only at 24 h following Vip3Aa treatment, is available in Table S3.

**Figure 4:**
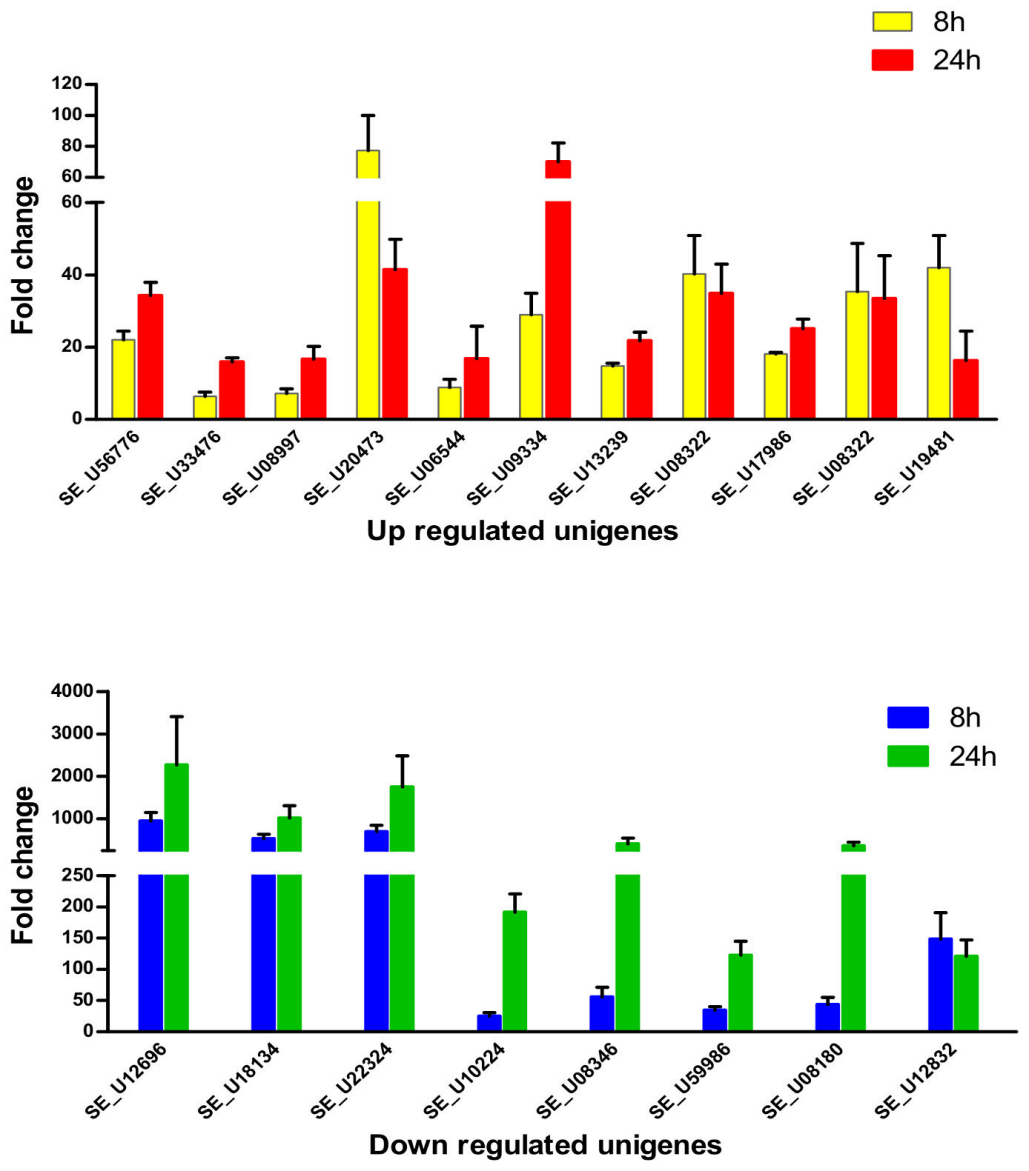
Validation of microarray data by qRT-PCR. The fold-change values (2^-ΔΔCt^) represent the mean and standard error of at least three replicates. The down-regulated fold-change values shown are the inverse of the 2^-ΔΔCt^ values.

The microarray data were validated by qRT-PCR. From the most strongly regulated, we chose eleven up-regulated and eight down-regulated unigenes (underlined unigenes in Table 1) to confirm the microarray results. The validation was also performed for *repat1* (unigene Se_U19481), since it has been reported to be involved in the response of *S. exigua* to Bt Cry intoxication [29] and, in the present study, was found specifically regulated after 8 h of Vip3Aa feeding exhibiting 41-fold up-regulation (Table S3). The up-regulated genes selected for the validation included homologous to genes involved in response to pathogens or defense, transport of proteins and metabolism. The down-regulated genes selected for validation included the three unigenes most strongly repressed (coding for unknown proteins), and unigenes homologous to genes coding for peritrophic matrix proteins and metabolism related enzymes. The qRT-PCR validation results are summarized in Figure 4. The expression values obtained by qRT-PCR confirmed the microarray results at 8 h and 24 h, thus confirming their respective profiles of expression over time. A comparison of the mean values of expression obtained from the microarray and from qRT-PCR is shown in Figure S2.

**Table 1:** *S. exigua* unigenes with the highest levels of regulation after Vip3Aa feeding.

**Unigene**	Target Name*	**Fold change 8 h**	**p-value**	**Fold change 24 h**	**p-value**	**Name**	**Blastx First Hit**	**E-value**
SE_U11312	SEX-LxN-C3620 sense	18.60	1.57E-03	163.03	1.72E-04	REPAT23	gi|383931963|gb|AFH57143.1| repat 23 [Spodoptera exigua]	3.00E-92
SE_U56776	SEX-LxN-C4302	51.14	2.68E-03	78.52	1.86E-03	juvenile hormone binding protein	gi|255977200|dbj|BAH97092.1| juvenile hormone binding protein [Bombyx mori]	4.44E-32
SE_U13520	SEX-LxN-C13620_sense	16.88	1.20E-02	72.68	2.08E-03	diapausin precursor	gi|156891151|gb|ABU96713.1| diapausin precursor [Spodoptera litura]	3.27E-19
SE_U33476	SEX-LxN-C795	22.95	6.17E-04	44.87	1.32E-04	diapausin precursor	gi|156891151|gb|ABU96713.1| diapausin precursor [Spodoptera litura]	2.16E-18
SE_U08997**	SEX-LxN-C2890	18.70	1.44E-03	43.77	5.48E-03	The regulation of *Heliothis virescens* innate immune responses by parasitization of *Campoletis sonorensis*	No hits found	2E-09 (Blastn)
SE_U20473	SEX-LxN-C14410	73.13	1.16E-03	40.53	1.96E-03	pancreatic lipase	gi|187884606|gb|ACD37364.1| pancreatic lipase 2 [Mamestra configurata]	1.66E-70
SE_U06544**	SEX-LxN-C2345	15.58	2.13E-03	35.17	7.86E-03	The regulation of *Heliothis virescens* innate immune responses by parasitization of *Campoletis sonorensis*	No hits found	2E-09 (Blastn)
SE_U09334	SEX-LxN-C111	16.38	1.58E-03	36.28	2.12E-04	REPAT2	gi|209868384|gb|ACI90727.1|Repat2 [Spodoptera exigua]	3.00E-87
SE_U13239	SEX-LxN-C4303	20.16	1.43E-04	30.99	8.98E-05	juvenile hormone binding protein	gi|255977200|dbj|BAH97092.1| juvenile hormone binding protein [Bombyx mori]	1.05E-17
SE_U08322	SEX-LxN-C13100	27.79	2.84E-03	30.43	2.88E-03	pancreatic lipase	gi|187884606|gb|ACD37364.1| pancreatic lipase 2 [Mamestra configurata]	1.66E-70
SE_U17986	SEX-LxN-C18774	35.10	1.14E-03	28.09	1.31E-02	juvenile hormone binding protein	gi|255977200|dbj|BAH97092.1| juvenile hormone binding protein [Bombyx mori]	2.00E-74
SE_U13929	SEX-LxN-C16964_sense	10.66	1.55E-02	27.16	5.49E-05	glutathione s-transferase 2	gi|112983028|ref|NP_001037077.1| glutathione S-transferase 2 [Bombyx mori]	8.32E-50
SE_U18528	SEX-LxN-C7876	5.37	9.19E-03	25.04	8.16E-04	phosphoenolpyruvate carboxykinase	gi|95103060|gb|ABF51471.1| mitochondrial phosphoenolpyruvate carboxykinase isoform 4 [Bombyx mori]	1.73E-149
SE_U02497	SEX-LxN-C12451	16.89	2.15E-04	24.82	1.68E-04	insulin-related peptide binding protein	gi|7407187|gb|AAF61949.1|AF236641_1 insulin-related peptide binding protein [Spodoptera frugiperda]	1.04E-93
SE U08322	SEX-LxN-C9182	23.27	4.12E-03	24.36	4.81E-03	pancreatic lipase	gi|187884606|gb|ACD37364.1| pancreatic lipase 2 [Mamestra configurata]	1.79E-08
SE_U10027	SEX-LxN-C3563_sense	12.28	2.52E-03	23.61	2.99E-04	diapausin precursor	gi|156891151|gb|ABU96713.1| diapausin precursor [Spodoptera litura]	5.70E-19
SE_U20685	SEX-LxN-C17485	14.29	1.26E-03	22.21	6.80E-04	REPAT2	gi|209868384|gb|ACI90727.1| REPAT2 [Spodoptera exigua]	3.00E-46
SE_U20782	SEX-LxN-C2522	12.74	8.52E-04	20.02	5.00E-81	REPAT16	gi|383931949|gb|AFH57136.1| Repat16 [Spodoptera exigua]	4.00E-82
SE_U20516	SEX-LxN-C1716	5.18	4.95E-03	19.74	1.40E-03	lebocin-like protein	gi|171262319| gb |ACB45566.1| lebocin-like protein [Antheraea pernyi]	2.00E-23
SE_U22077	SEX-LxN-C16929_sense	17.50	3.04E-04	18.46	2.98E-04	putative cuticle protein	gi|223671103|tpd|FAA00503.1| TPA: putative cuticle protein [Bombyx mori]	8.85E-83
SE_U57703	SEX-LxN-C3107	17.21	2.70E-04	17.42	3.12E-04	cuticle protein 3	gi|56462130|gb|AAV91348.1| cuticle protein 3 [Lonomia obliqua]	2.87E-23
SE_U08322	SEX-LxN-C7110	15.10	5.17E-03	17.25	5.85E-03	pancreatic lipase	gi|187884606|gb|ACD37364.1| pancreatic lipase2 [Mamestra configurata]	1.66E-70
SE_U18692	SEX-LxN-C2625	-6.37	6.93E-04	-33.85	1.29E-03	chymotrypsin-like precursor	gi|255046231|gb|ACU00133.1| chymotrypsin-like protein precursor [Spodoptera litura]	1.71E-100
SE_U39115	SEX-LxN-C696	-5.35	3.68E-03	-34.57	6.67E-04	lipase	gi|171740897|gb|ACB54943.1| lipase [Helicoverpa armigera]	5.15E-04
SE_U16836	SEX-LxN-C4227	-32.61	2.69E-05	-36.76	1.00E-05	acyl-CoA oxidase	gi|357602837|gb| EHJ63527.1| putative acyl-CoA oxidase [Danaus plexippus]	5.00E-116
SE_U14733	SEX-LxN-C6000	-10.06	1.36E-03	-38.24	6.67E-05	serine protease	gi|304443637|gb|ACR16002.2| serine protease 48 [Mamestra configurata]	2.40E-94
SE_U10377	SEX-LxN-C4998	-4.35	3.16E-03	-40.28	1.36E-04	REPAT43	gi|383932003|gb|AFH57163.1|Repat43 [Spodoptera exigua]	4.00E-60
SE_U08232	SEX-LxN-C15193	-19.82	3.17E-03	-42.36	2.30E-03	cytochrome p450 monooxygenase	gi|21552587|gb|AAM54723.1| cytochrome P450 monooxygenase CYP4M7 [Helicoverpa zea]	8.12E-56
SE_U36716	SEX-LxN-C1692	-3.08	3.91E-02	-44.14	4.43E-04	chymotrypsin-like protein	gi|300680014 |gb|ADK27715.1|chymotrypsin-like protein 2 [Spodoptera litura]	2.00E-160
SE_U14607	SEX-LxN-C2974	-37.60	9.16E-04	-46.21	5.84E-04	pancreatic lipase	gi|187884606|gb|ACD37364.1| pancreatic lipase 2 [Mamestra configurata]	3.75E-43
SE_U19812	SEX-LxN-C18571_sense	-21.08	5.60E-03	-53.33	4.93E-03	cytochrome P450 monooxygenase	gi|21552587|gb|AAM54723.1| cytochrome P450 monooxygenase CYP4M7 [Helicoverpa zea]	3.63E-108
SE_U09066	SEX-LxN-C881	-7.69	1.11E-02	-54.09	2.19E-04	serine protease	gi|304443603|gb|ACR15971.2|serine protease 37 [Mamestra configurata]	3.00E-97
SE_U13832	SEX-LxN-C3291	-8.17	1.01E-02	-54.42	3.46E-06	1 3-dehydroecdysone 3alpha-reductase	gi|7862150|gb|AAF70499.1|AF255341_1 3-dehydroecdysone 3alpha-reductase [Spodoptera littoralis]	3.76E-41
SE_U12832	SEX-LxN-C17214	-59.83	6.74E-04	-69.82	2.13E-04	pancreatic lipase	gi|187884606|gb|ACD37364.1| pancreatic lipase 2 [Mamestra configurata]	1.14E-85
SE_U08232	SEX-LxN-C17101_sense	-24.36	9.22E-04	-74.24	1.48E-04	cytochrome P450 monooxygenase	gi|21552587|gb|AAM54723.1| cytochrome P450 monooxygenase CYP4M7 [Helicoverpa zea]	3.63E-108
SE_U57515	SEX-LxN-C371	-10.88	1.42E-03	-78.06	2.94E-03	chymotrypsinogen	gi|8037819|gb|AAF71517.1|AF233730_1 AiC6 chymotrypsinogen [Agrotis ipsilon]	1.58E-21
SE_U13695	SEX-LxN-C2240	-12.49	1.45E-03	-88.83	3.45E-03	chymotrypsin precursor	gi|29501764|gb|AAO75039.1| chymotrypsin precursor [Spodoptera frugiperda]	2.95E-83
SE_U08180	SEX-LxN-C14967	-14.14	3.04E-03	-125.79	3.28E-04	chitin deacetylase	gi|187884602|gb|ACD37362.1| chitin deacetylase 1 [Mamestra configurata]	5.74E-58
SE_U59986	SEX-LxN-C20053	-26.83	2.07E-03	-135.34	1.61E-04	serine protease	gi|237700841|gb|ACR16003.1| serine protease 44 [Mamestra configurata]	3.88E-07
SE U08346	SEX-LxN-C18212	-12.85	3.05E-03	-138.02	3.18E-05	chitin deacetylase	gi|187884602|gb|ACD37362.1| chitin deacetylase 1 [Mamestra configurata]	5.37E-115
SE_U10224	SEX-LxN-C13563	-16.60	3.53E-03	-207.28	2.64E-04	chitin deacetylase	gi|187884602|gb|ACD37362.1| chitin deacetylase 1 [Mamestra configurata]	5.74E-58
SE_U08783	SEX-LxN-C3645	-6.84	7.76E-03	-223.65	3.49E-04	chymotrypsin-like protease	gi|151199948|gb|ABR88231.1| chymotrypsin-like protease C1 [Heliothis virescens]	1.41E-84
SE_U22324**	SEX-LxN-C19458	-333.50	1.47E-04	-332.38	5.77E-04	No hits found	No hits found	--
SE_U18134**	SEX-LxN-C16094	-298.95	2.48E-04	-480.01	9.06E-05	No hits found	No hits found	--
SE_U12696**	SEX-LxN-C1037	-267.99	1.00E-04	-656.79	4.31E-04	No hits found	No hits found	--

The Table shows the 20 more up-regulated and the 20 more down-regulated unigenes that show homology to proteins in public databases, extracted from the list of regulated unigenes at both 8 and 24 h of treatment with Vip3Aa. Data are presented in descending order according to the expression values at 24 h. Unigenes which expression has been validated by qRT-PCR are underlined.

* Target Name represents the mean from two probes, unless “sense” or “anti” is indicated in which case only one probe is represented.

** Sequences with no protein homologous in databases but highly regulated or related with lepidopteran immune reaction.

To determine the type of biological processes and pathways that were affected by Vip3Aa intoxication, the functional clustering toolbox DAVID v.6.7 was used. The analysis resulted in the identification of 21 functional clusters. Table 2 summarizes the clusters with enrichment scores higher than 0.50 (the remaining clusters are reported in Table S4). The cluster with highest scoring (Cluster 1) included genes encoding hormone-binding, odorant binding and juvenile hormone-binding proteins. Homologous genes had been also found regulated in studies on *Spodoptera frugiperda* [31], *Choristoneura fumiferana* [30] and *B.mori* [56] intoxicated with Cry1Ca, Cry1Ab or *Bacillus bombyseptieus*, respectively. Recently, it was found that an odorant binding protein (related to the immune system response) in the coleopteran *Tribolium castaneum*, was specifically overexpressed after exposure to toxic Cry proteins [37]. 

**Table 2:** Functional annotation clusters of genes regulated by Vip3Aa treatment.

**Annotation Cluster**	**Term**	**Nr of genes in this cluster**	**% of genes in this cluster**	**p-value**
Cluster 1	Enrichment Score: 2.09			
	IPR013053:Hormone binding	5	0.52	6.97E-03
	IPR004272:Odorant binding protein	5	0.52	1.08E-02
	SM00700:JHBP	5	0.52	1.17E-02
Cluster 2	Enrichment Score: 1.18			
	IPR001304:C-type lectin	4	0.42	4.71E-02
	IPR016186:C-type lectin-like	4	0.42	4.71E-02
	SM00034:CLECT	4	0.42	5.08E-02
	Lectin	4	0.42	6.29E-02
	IPR018378:C-type lectin, conserved site	3	0.31	1.74E-01
Cluster 3	Enrichment Score: 1.03			
	IPR012674:Calycin	3	0.31	7.53E-02
	IPR000566:Lipocalin-related protein and Bos/Can/Equ allergen	3	0.31	7.53E-02
	GO:0008289~lipid binding	4	0.42	1.43E-01
Cluster 4	Enrichment Score: 0.84			
	IPR003598:Immunoglobulin subtype 2	3	0.31	1.06E-01
	IPR013098:Immunoglobulin I-set	3	0.31	1.06E-01
	SM00408:IGc2	3	0.31	1.12E-01
	IPR007110:Immunoglobulin-like	3	0.31	1.74E-01
	IPR013783:Immunoglobulin-like fold	3	0.31	2.85E-01
Cluster 5	Enrichment Score: 0.72			
	GO:0031090~organelle membrane	4	0.42	1.47E-01
	GO:0005743~mitochondrial inner membrane	3	0.31	1.60E-01
	GO:0019866~organelle inner membrane	3	0.31	1.60E-01
	GO:0031966~mitochondrial membrane	3	0.31	1.60E-01
	GO:0005740~mitochondrial envelope	3	0.31	1.76E-01
	GO:0031975~envelope	3	0.31	1.93E-01
	GO:0031967~organelle envelope	3	0.31	1.93E-01
	GO:0044429~mitochondrial part	3	0.31	2.10E-01
	GO:0005739~mitochondrion	3	0.31	4.16E-01
Cluster 6	Enrichment Score: 0.67			
	IPR002018:Carboxylesterase, type B	5	0.52	1.17E-01
	IPR019826:Carboxylesterase type B, active site	4	0.42	1.68E-01
	Lipid metabolism	4	0.42	4.88E-01
Cluster 7	Enrichment Score: 0.51			
	GO:0055114~oxidation reduction	14	1.46	3.67E-02
	GO:0046872~metal ion binding	21	2.19	7.22E-02
	GO:0043169~cation binding	21	2.19	1.12E-01
	GO:0043167~ion binding	21	2.19	1.12E-01
	metal-binding	11	1.15	2.12E-01
	iron	7	0.73	2.66E-01
	GO:0005506~iron ion binding	8	0.83	2.95E-01
	heme	5	0.52	3.75E-01
	GO:0020037~heme binding	5	0.52	4.43E-01
	GO:0046906~tetrapyrrole binding	5	0.52	4.43E-01
	GO:0046914~transition metal ion binding	13	1.36	4.95E-01
	GO:0009055~electron carrier activity	6	0.63	5.02E-01
	oxidoreductase	8	0.83	5.21E-01
	Monooxygenase	4	0.42	5.36E-01
	Secondary metabolites biosynthesis, transport, and catabolism	5	0.52	5.50E-01
	IPR002401:Cytochrome P450, E-class, group I	4	0.42	5.61E-01
	IPR017972:Cytochrome P450, conserved site	4	0.42	5.61E-01
	IPR017973:Cytochrome P450, C-terminal region	4	0.42	6.06E-01
	IPR001128:Cytochrome P450	4	0.42	6.06E-01

The second cluster consisted of C-type lectins and lectin-like proteins, which are sugar binding proteins involved in biological recognition pathways that are involved in the immune system [21,59,60]. The third cluster was comprised of lipocalin-related proteins, which are transporters of small hydrophobic proteins involved in many biological processes like the immune response and pheromone transport. The remaining clusters included proteins involved in pattern recognition, such as immunoglobulin-like proteins (Cluster 4); and proteins involved in biosynthesis, transport, and metabolism, such as carboxylesterases, ion binding proteins, cytochrome P450 and redox proteins (Clusters 6, 9, 10, 13, 14, 15, and 7, that grouped the redox reaction enzymes and that is the cluster with more genes represented). Other clusters consisted of proteinase inhibitors and proteolytic enzymes (Clusters 12 and 20), mitochondrial envelope proteins (Cluster 5) cytoskeletal structural proteins (Cluster 16), integral membrane proteins (Cluster 8), proteins involved in nucleic acid biosynthetic processes (Cluster 11), and proteins involved in the regulation of transcription and translation (Clusters 17, 18, 19 and 21).

**Figure 5:**
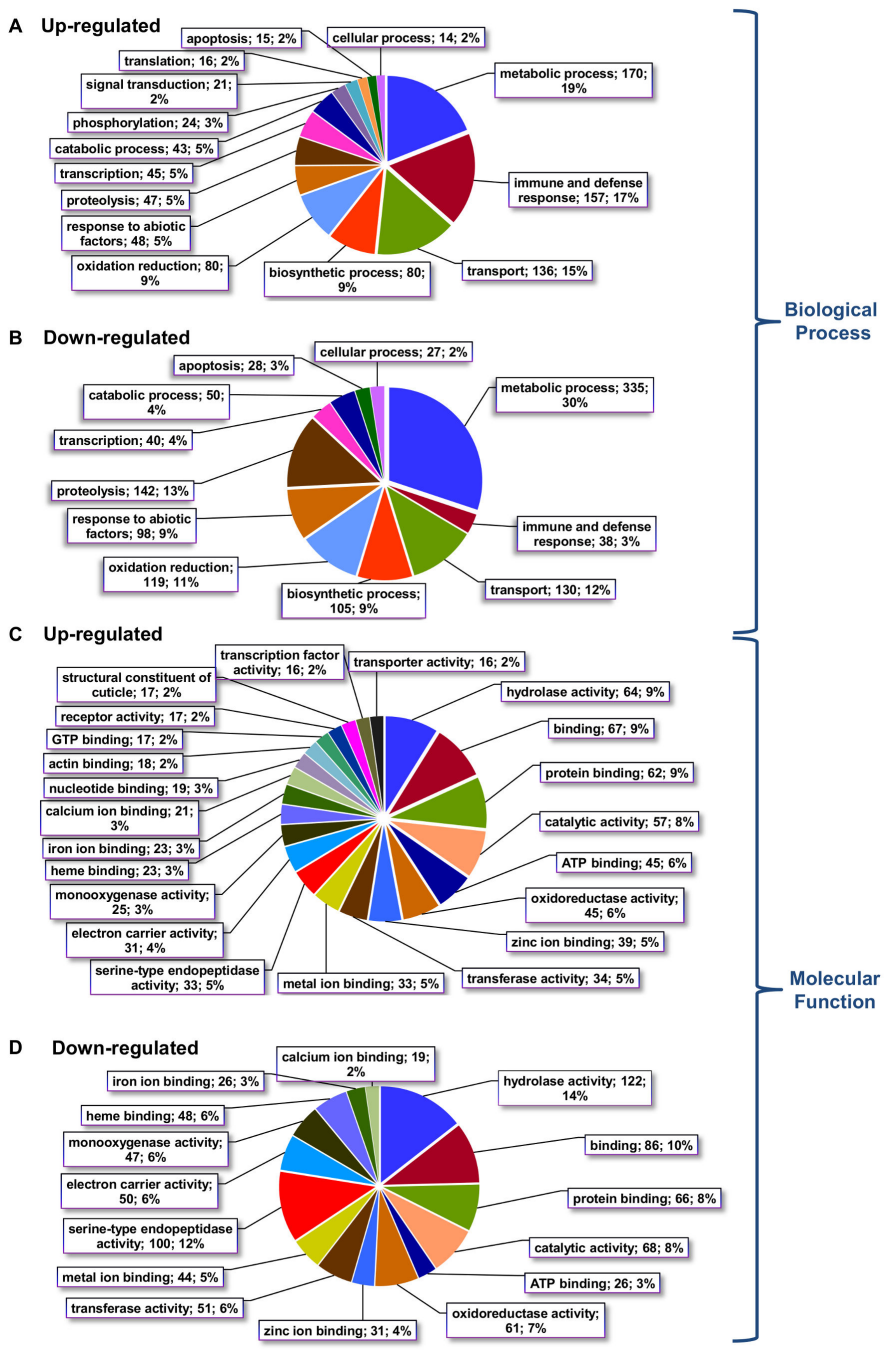
Gene Ontology assignments (level 3) for the S. *exigua* midgut unigenes regulated by Vip3Aa feeding. Gene Ontology (GO) assignments for up-regulated and down-regulated unigenes as predicted for their involvement in the Biological Processes (A and B respectively) and Molecular Functions (C and D respectively) categories. The diagrams only show groups that have at least 1% represented members.

We also determined the Gene Ontology (GO) term assignments for the up-regulated and the down-regulated unigenes in the Biological Process and the Molecular Function domains, at level 3 (Figure 5). To simplify, only classes represented by more than 1% of the total amount of regulated sequences were included. At the Biological Process domain (Figures 5A and 5B), one of the most noteworthy differences between the up- and down-regulated unigenes was observed in the “metabolic process” category, the most abundant in the S. *exigua* transcriptome [42], which had about twice as many representatives in the down-regulated genes (335) than in the up-regulated genes (170). Consistently, at the Molecular Function domain, the most represented category was “hydrolase activity” in both up- and down-regulated groups, as had also been detected in the transcriptome analysis of lepidopteran immune-activated larvae [42,61], but with the particularity that we observed more assigned unigenes in the down-regulated sequences (Figure 5C and 5D). The same observation was found for the “serine-type endopeptidase activity” or “monooxygenase activity” categories. The down-regulation of these processes could be a consequence of the deceleration of metabolism after Vip feeding, and is likely linked to the high growth inhibition effect (99%) and feeding cessation. This is in agreement with genome-wide gene expression analysis performed on insect midguts after Cry toxin or bacterial feeding, which point to a general down-regulation of digestive proteins, such as lipases or proteases [20,30,31,34,37].

Interestingly, at the Biological Processes domain, a large difference was observed between the distribution of up- and down-regulated genes in the “immune and defense response” category (157 genes versus 38 genes, Figures 5A and 5B). These results agree with previous gene expression studies, which showed the up-regulation of genes involved in detoxification, stress response, immune system and epithelial renewal, after bacterial infection or toxin challenge [20,30,34]. Consistent with these GO analyses, an overview of the regulated unigenes (listed in Table S3) shows a distribution of roles for the up- and down-regulated genes. Table 1 summarizes the 20 most induced and the 20 most repressed unigenes at both treatment times. The strongest up-regulated unigenes included genes encoding immune-related and response to abiotic-factors (mainly from the *repat* family), hormone modulation (e.g. JH binding protein), and detoxification (e.g. glutathione S-transferase) proteins. The strongly repressed unigenes were mainly genes encoding digestive enzymes (e.g. serine proteases) and proteins involved in oxidoreductase reactions (e.g. cytochrome P450). Interestingly, among the most down-regulated genes we found chitin deacetylase, an enzyme that increases the permeability of the gut peritrophic membrane (PM) [62]. This enzyme was also down-regulated in *Helicoverpa armigera* and in *S. exigua* after baculovirus infection, and this regulation was explained as a mechanism of the insect to reduce its susceptibility to the ingested pathogen by decreasing PM permeability [62]. A similar mechanism could decrease the amount of Vip3A toxin passing through the PM and binding to the midgut epithelial cells. However, up-regulation of this type of enzymes was described in the coleopteran *Tenebrio molitor* intoxicated with Cry3A protoxin [34]. This could be due to differences between lepidopterans and coleopterans, since the latter can survive Cry intoxication for weeks without obvious signs of paralysis [34], or to the biological role of each chitin deacetylase protein.

In addition to the 40 most regulated unigenes, Table 1 also includes three unigenes of unknown function that exhibit the strongest values of down-regulation found in this study (from 332-fold to 657-fold, at 24 h). These three unigenes were further investigated and manually assembled into a single contig of 672 nucleotides (GeneBank Acc. No. KF601929), which showed homology to a *B. mori* gene and a *H. armigera* EST. Alignment of the putative encoded proteins (named REVIP in *S. exigua* because was detected in REsponse-to-Vip intoxication) is shown in Figure S3. No homologues of this protein were found in other insect orders.

### Immune-related genes regulated after Vip3Aa ingestion

 The S. *exigua* larvae transcriptome represented in the microarray was specifically aimed to be enriched for pathogen-related genes and, therefore, offers the potential to detect variations in expression level of many immune-related genes and pathways with greater accuracy. When describing the larval transcriptome, Pascual et al. [42] divided the immune-related genes into three categories: (a) genes involved in pathogen recognition, (b) genes coding for components of the main immune-related signaling pathways (Toll, IMD, JAK/STAT, and p38 MAPK) and melanization processes, and (c) genes of antimicrobial effectors induced by these immune-activated pathways. In the present study, the regulation of such genes, and of other immune-related genes (such as the AMP spodoptericin or unigenes homologous to the lepidopteran immune-related genes *Hdd11* and *Hdd23*), after Vip3Aa intoxication has been screened. The results are summarized in Table 3.

**Table 3:** Immune-related unigenes regulated by Vip3Aa feeding.

**Gene name**	**Function**	**Unigene**	Target Name*	**First Hit**	**Fold change 8 h**	**p-value 8 h**	**Fold change 24 h**	**p-value 24 h**
*PGRP*	Recognition	SE_U09954**	SEX-LxN-C988	gi|18202160|gb| O76537.1 |PGRP_TRINI RecName: Full=Peptidoglycan recognition protein; Flags: Precursor [Trichoplusia ni ]	2.29	1.14E-02	2.94	1.81E-02
		SE_U21489	SEX-LxN-C17630	gi|399128032|bg|JX082167.1| Helicoverpa armigera peptidoglycan recognition protein C (PGRP-C)	-9.14	6.26E-04	-5.80	1.07E-02
		SE_U35288**	SEX-LxN-C18688	gi|113208234|dbj|BAF03521.1| peptidoglycan recognition protein C [Samia cynthia ricini]	-12.29	4.94E-04	-6.49	7.30E-03
		SE_U21399	SEX-LxN-C574	gi|113208232|dbj|BAF03520.1| peptidoglycan recognition protein B [Samia cynthia ricini]	-2.35	1.68E-02	-1.21	2.63E-01
		SE_U21524	SEX-LxN-C5919	gi|357616833|ref|EHJ70432.1| peptidoglycan recognition protein S2 [Danaus plexippus]	-2.39	3.72E-02	-1.02	4.87E-01
		SE_U21904	SEX-LxN-C1186	gi|113208232|dbj|BAF03520.1| peptidoglycan recognition protein B [Samia cynthia ricini]	-4.01	1.35E-02	-1.75	1.97E-01
		SE_U04294	SEX-LxN-C362	gi|113208232|dbj|BAF03520.1| peptidoglycan recognition protein B [Samia cynthia ricini]	-5.98	1.43E-02	-2.01	2.02E-01
*ßGRP*	Recognition	SE_U11347	SEX-LxN-C1710	gi|208972529|gb|ACI32825.1| beta-1,3-glucan recognition protein 1 [Helicoverpa armigera]	2.41	2.80E-03	3.30	8.51E-04
		SE_U00225	SEX-LxN-C16411	gi|208972531|gb|ACI32826.1| beta-1,3-glucan recognition protein 2a [Helicoverpa armigera]	1.55	1.74E-03	3.04	2.50E-03
		SE_U20847**	SEX-LxN-C2788	gi|208972531|gb|ACI32826.1| beta-1,3-glucan recognition protein 2a [Helicoverpa armigera]	1.53	4.93E-04	3.00	1.50E-03
		SE_U09598	SEX-LxN-C1305	gi|52782739| gb |Q8ISB6.1| RecName: Full=Beta-1,3-glucan-binding protein 2; Short=BGBP-2; AltName: Full=Beta-1,3-glucan recognition protein 2; Short=BetaGRP-2; Flags: Precursor	1.43	1.25E-02	2.77	2.78E-03
		SE_U15491	SEX-LxN-C365	gi|3506272423|gb|AEQ33590.1| beta-1,3-glucan recognition protein 2 [Spodoptera litura]	1.36	3.42E-01	4.46	3.40E-02
		SE_U05118	SEX-LxN-C2375	gi|208972535|gb|ACI32828.1| beta-1,3-glucan recognition protein 3 [Helicoverpa armigera]	-2.12	1.12E-02	-1.36	1.39E-01
		SE_U03464	SEX-LxN-C8076	gi|208972535|gb|ACI32828.1| beta-1,3-glucan recognition protein 3 [Helicoverpa armigera]	-2.16	8.29E-03	-1.39	1.08E-01
		SE_U24587	SEX-LxN-C3511	gi|208972535|gb|ACI32828.1| beta-1,3-glucan recognition protein 3 [Helicoverpa armigera]	-2.29	6.53E-03	-1.41	7.01E-02
*Scavenger receptor*	Recognition	SE_U00476	SEX-LxN-C8180	gi|91080607|ref| XP_967476.1 | similar to scavenger receptor cysteine-rich protein isoform 1 [Tribolium castaneum]	2.80	1.83E-03	2.65	3.28E-03
		SE_U18456	SEX-LxN-C3077	gi|91080607|ref|XP_967476.1|PREDICTED: similar to scavenger receptor cysteine-rich protein isoform 1 [Tribolium castaneum]	2.24	9.23E-02	4.01	2.93E-02
*lectin*	Recognition	SE_U20725	SEX-LxN-C16050	gi|385202645|gb|AFI47448.1| C-type lectin 3 [Helicoverpa armigera]	2.55	1.84E-02	4.62	6.57E-03
		SE_U13809	SEX-LxN-C13991	gi|114052520|ref|NP_001040251.1| lectin 4 C-type lectin [Bombyx mori]	3.43	1.82E-03	3.23	4.43E-03
		SE_U15570**	SEX-LxN-C3779	gi|238915971|gb|ACR78452.1| C-type lectin [Heliothis virescens]	2.56	5.93E-03	2.83	1.08E-02
		SE_U05936	SEX-LxN-C14021	gi|87248169|gb|ABD36137.1| lectin 5 [Bombyx mori]	-2.94	7.93E-03	-3.01	7.32E-03
		SE_U17978	SEX-LxN-C1682	gi|195963381|ref|NP_001124371.1| low-expression lectin 1 [Bombyx mori]	-3.36	1.23E-02	-4.63	3.65E-03
		SE_U49052	SEX-LxN-C14022	gi|56462354|gb|AAV91460.1| lectin 5 [Lonomia obliqua]	-24.70	7.88E-04	-30.54	1.64E-03
*Spatzle*	Toll pathway member	SE_U25489	SEX-LxN-C11313	gi|389608503|gb|BAM17861.1|spatzle 3 [Papilio xuthus]	-4.61	9.04E-03	-5.47	1.15E-03
*PPAE*	Melanization	SE_U16717	SEX-LxN-C4232	gi|56718390|gb|AAW24481.1| prophenol oxidase activating enzyme 3 [Spodoptera litura]	3.51	1.52E-05	5.05	1.01E-06
		SE_U21232	SEX-LxN-C18670	gi|56718388|gb|AAW24480.1| prophenol oxidase activating enzyme 1 [Spodoptera litura]	3.45	4.59E-03	4.37	4.88E-04
		SE_U56479	SEX-LxN-C19306	gi|56718388|gb|AAW24480.1| prophenol oxidase activating enzyme 1 [Spodoptera litura]	3.35	1.28E-03	4.13	2.45E-04
		SE_U11184	SEX-LxN-C12171	gi|56718390|gb|AAW24481.1| prophenol oxidase activating enzyme 3 [Spodoptera litura]	2.25	1.88E-02	3.23	8.07E-03
		SE_U01310	SEX-LxN-C11024	gi|56718390|gb|AAW24481.1| prophenol oxidase activating enzyme 3 [Spodoptera litura]	1.93	6.50E-03	2.49	2.13E-03
*serpin*	Melanization	SE_U15354	SEX-LxN-C15300	gi|160333383|ref|NP_001103823.1| serpin-6 [Bombyx mori] gi|157786102|gb|ABV74209.1| serpin-6 [Bombyx mori]	12.00	5.26E-04	6.01	1.50E-03
		SE_U18684	SEX-LxN-C18484	gi|226342880|ref|NP_001139702.1| serpin 8 [Bombyx mori] gi|195972022|gb|ACG61173.1| serpin-8 [Bombyx mori]	3.89	1.30E-03	5.48	3.02E-03
		SE_U02392	SEX-LxN-C17615	gi|156254836|gb|ABU62829.1| serpin-2 [Spodoptera exigua]	2.89	5.46E-05	2.48	5.23E-04
		SE_U13385	SEX-LxN-C17616	gi|156254836|gb|ABU62829.1| serpin-2 [Spodoptera exigua]	2.95	8.61E-06	2.42	3.86E-03
		SE_U11456**	SEX-LxN-C14805	gi|195972030|gb|ACG61177.1| serpin-14 [Bombyx mori]	2.25	7.51E-03	2.34	1.23E-02
		SE_U09976**	SEX-LxN-C17074	gi|270358644|ref|ACZ81437.1| serpin-4 [Bombyx mori]	1.79	1.60E-02	2.41	6.05E-03
		SE_U13244**	SEX-LxN-C17218	gi|112984548|ref|NP_001037205.1| serpin-5 [Bombyx mori]	-2.23	1.22E-02	-2.69	2.06E-02
		SE_U03454	SEX-LxN-C3936	gi|226342880|ref|NP_001139702.1| serpin 8 [Bombyx mori]	1.45	1.36E-01	2.90	2.39E-02
		SE_U24322	SEX-LxN-C6873	gi|45594226|gb|AAS68504.1| serpin-4B [Manduca sexta]	1.71	5.59E-02	2.17	5.23E-03
		SE_U10611	SEX-LxN-C18279	gi|226342920|ref|NP_001139722.1| serpin 31 [Bombyx mori]	-1.24	2.66E-01	2.08	4.03E-02
		SE_U20987**	SEX-LxN-C16848	gi|226342878|ref|NP_001139701.1| serpin 7 [Bombyx mori]	-2.12	7.97E-02	-3.26	1.29E-02
		SE_U21680**	SEX-LxN-C18616	gi|226342878|ref|NP_001139701.1| serpin 7 [Bombyx mori]	-2.23	5.98E-02	-3.54	1.59E-02
		SE_U02069	SEX-LxN-C19090	gi|226342878|ref|NP_001139701.1| serpin 7 [Bombyx mori]	-2.35	6.44E-02	-3.97	1.32E-02
*cecropin*	Effector	SE_U08141	SEX-LxN-C1119	gi|46396048|sp|Q9XZH0.1|CECB_SPOLT RecName: Full=Cecropin-B; Flags: Precursor	1.38	6.75E-02	3.18	1.34E-02
		SE_U15514	SEX-LxN-C15308	gi|147744339|gb|ABQ51092.1| cecropin D [Spodoptera litura]	1.17	3.71E-01	2.99	3.95E-02
*attacin*	Effector	SE_U12714	SEX-LxN-C16429	gi|363497923|gb|AEW24425.1| attacin [Spodoptera exigua]	2.54	2.03E-02	3.73	1.93E-02
		SE_U08212	SEX-LxN-C18887	gi|363497923|gb|AEW24425.1| attacin [Spodoptera exigua]	2.60	2.48E-02	3.63	2.23E-02
		SE_U18185**	SEX-LxN-C2524	gi|238915975|gb|ACR78454.1| attacin [Heliothis virescens]	2.66	2.27E-02	3.58	2.67E-02
*gloverin*	Effector	SE_U10179	SEX-LxN-C3153	gi|302347126|gb|ADL27731.1|gloverin [Spodoptera exigua]	3.51	1.53E-03	9.03	8.16E-04
*diapausin*	Effector	SE_U33476	SEX-LxN-C795	gi|156891151|gb|ABU96713.1| diapausin precursor [Spodoptera litura]	22.95	6.17E-04	44.87	1.32E-04
		SE_U14458	SEX-LxN-C1257	gi|156891151|gb|ABU96713.1| diapausin precursor [Spodoptera litura]	1.79	8.14E-02	9.69	1.25E-03
		SE_U22863	SEX-LxN-C19788	gi|156891151|gb|ABU96713.1| diapausin precursor [Spodoptera litura]	3.66	5.03E-02	6.77	2.49E-02
*lebocin*	Effector	SE_U20516	SEX-LxN-C1716	gi|171262319| gb |ACB45566.1| lebocin-like protein [Antheraea pernyi]	5.18	4.95E-03	19.74	1.40E-03
*moricin*	Effector	SE_U21407	SEX-LxN-C956	gi|33146300|gb|BAC79440.1| moricin [Spodoptera litura]	1.46	4.55E-02	3.78	8.30E-03
		SE_U02249	SEX-LxN-C20207	gi|47834350|gb|AAT38873.1| moricin [Spodoptera exigua]	1.70	6.76E-03	4.58	3.33E-03
*cobatoxin*	Effector	SE_U53778	SEX-LxN-C2301	gi|33439724|gb|AAQ18900.1| cobatoxin long form B [Spodoptera frugiperda]	1.69	1.56E-02	6.35	1.22E-06
		SE_U18253	SEX-LxN-C18751	gi|33439724|gb|AAQ18900.1| cobatoxin long form B [Spodoptera frugiperda]	1.65	1.15E-02	6.28	7.93E-07
		SE_U14423	SEX-LxN-C20071	gi|33439724|gb|AAQ18900.1| cobatoxin long form B [Spodoptera frugiperda]	1.59	2.05E-02	5.97	5.34E-06
*lysozyme*	Effector	SE_U14399	SEX-LxN-C16831_sense	gi|357626014|gb|EHJ76261.1| lysozyme-like protein 1 [Danaus plexippus]	2.69	5.00E-03	3.62	5.02E-04
		SE_U14068**	SEX-LxN-C5984_anti	gi|260765455|gb|ACX49765.1| lysozyme-like protein 1 [Manduca sexta]	2.48	3.54E-03	3.26	6.34E-04
		SE_U14081	SEX-LxN-C5984_sense	gi|145286562|gb|ABP52098.1| lysozyme-like protein 1 [Antheraea mylitta]	2.07	2.68E-03	3.05	4.31E-05
		SE_U24430	SEX-LxN-C9442	gi|281398208|gb|ADA67927.1| putative lysozyme [Bombyx mori]	2.25	2.25E-02	2.96	1.58E-02
		SE_U13750	SEX-LxN-C19611	gi|29893332|gb|AAP03061.1| lysozyme [Spodoptera exigua]	1.92	1.99E-02	3.32	6.28E-03
		SE_U13949	SEX-LxN-C2090	gi|29893332|gb|AAP03061.1| lysozyme [Spodoptera exigua]	1.79	7.06E-04	2.39	1.06E-02
		SE_U13790	SEX-LxN-C19103	gi|29893332|gb|AAP03061.1| lysozyme [Spodoptera exigua]	1.76	1.36E-03	2.38	1.24E-02
		SE_U13563	SEX-LxN-C2118	gi|29893332|gb|AAP03061.1| lysozyme [Spodoptera exigua]	1.78	4.91E-03	2.26	1.58E-02
*spodoptericin /defensin*	Effector	SE_U05013	SEX-LxN-C2205	gi|363497927|gb|AEW24427.1|defensin [Spodoptera exigua]	2.74	5.74E-03	6.26	4.33E-04
		SE_U57438	SEX-LxN-C1559	gi|363497927|gb|AEW24427.1|defensin [Spodoptera exigua]	2.57	3.37E-03	5.74	1.70E-05
		SE_U59058	SEX-LxN-C19990	gi|363497927|gb|AEW24427.1|defensin [Spodoptera exigua]	2.05	2.25E-02	4.98	1.55E-04
*Hdd23*	Immune response	SE_U20839	SEX-LxN-C3047	gi|4090970|gb|AAD09282.1| immune-related Hdd23 [Hyphantria cunea]	7.13	3.89E-05	15.30	3.46E-04
		SE_U20478	SEX-LxN-C3046	gi|4090970|gb|AAD09282.1| immune-related Hdd23 [Hyphantria cunea]	2.29	3.86E-03	5.02	2.83E-04
*Hdd11*	Immune response	SE_U12373	SEX-LxN-C16571	gi|74873244|sp|O96382.1|DFP11_HYPCU RecName: Full=Putative defense protein Hdd11; AltName: Full=Hyphantria differentially displayed gene 11; Flags: Precursor	2.55	1.29E-02	4.93	3.18E-04
		SE_U21714	SEX-LxN-C3305	gi|74873244|sp|O96382.1|DFP11_HYPCU RecName: Full=Putative defense protein Hdd11; AltName: Full=Hyphantria differentially displayed gene 11; Flags: Precursor	2.43	1.04E-02	4.26	2.32E-04

PGRP= Peptidoglycan recognition protein; ßGRP= β glucan recognition protein; PPAE=Prophenol oxydase activating enzyme.

* Target name represents the mean from two probes, or represents only one probe if “sense” or “anti” is indicated.

** Unigenes revealed by more than two targets. In these cases, the Target Name in the Table is the one with higher p-value at 24 h; fold change values shown are the media of the fold change values obtained for the different targets.

 As mentioned above, we have observed a general up-regulation of immune-related genes. Among the different types of genes belonging to this group, we have found two notable features: (a) genes involved in pathogen recognition, melanization, and antimicrobial effectors were regulated after Vip3Aa intoxication; and (b) genes encoding components of the main immune related signaling pathways, such as Toll, IMD, JAK/STAT, MAPK p38, and JNK MAPK pathways, were not regulated.

 We have not observed a clear pattern of regulation for the pathogen recognition proteins PGRP, ßGRP, lectins and hemolin in midguts. In lepidopteran hemolymph these proteins are constitutively present and trigger pathogen responses as phagocytosis, nodule formation, encapsulation, melanization and synthesis of AMPs [59,60]. In this study, after Vip3Aa feeding some unigenes homologous to PGRP precursor, ßGRP 1, 2a, and 2, and scavenger receptor and C-type lectins (carbohydrate recognition proteins that form Cluster 2 in Table 2) were only slightly overexpressed. However, unigenes with homology to lectins 1 and 5, PGRP-C and -B (to a lesser extent), and ßGRP3 were down-regulated, especially after 8 h treatment. Neither hemolin, nor Gram negative recognition proteins (GNRP) homologous genes, both present in the microarray, were found regulated in midguts after Vip3Aa intoxication.

 Melanization occurs regularly in the midgut and hindgut of lepidopterans such as *B. mori*, to regulate fecal microbiota [63], and is permanently induced in the hemolymph of Bt-tolerant strains of *Ephestia kuehniella* [64] and in Cry1Ac resistant strains of *H. armigera* [65]. In this work, we also observed the regulation (generally overexpression) of several genes encoding members of the melanization cascade after Vip3A intoxication. PPO activating enzyme, a necessary component for the initiation of the melanization pathway [60], was up-regulated. Serpins, proteinase inhibitors that regulate the serin protease cascade for melanization, as well as other immune proteinase pathways in insects [21], were also regulated, but in this case with different behavior according to their family. Serpins 2, 4, 14, 31 and, to a greater extent, 6 and 8 were up-regulated. This was similar to results observed for serpin 2 in *C. fumiferana* after sublethal Cry1Ab intoxication [30], and for serpin 4 in *Aedes aegypti* intoxicated with Cry11Aa [36], and in *S. frugiperda* intoxicated with Cry1Ca [31]. In contrast, serpins 5 and 7 were down-regulated. The up- and down-regulation of serpins was also found in *B. mori* intoxicated with *B. bombyseptieus* [56]. The function of the different serpins and their correlation with melanization processes, protein agglutination, and regulation of other immune related signaling pathways remains unclear [21].

 The clearest pattern of regulation of immune-related genes was observed for those genes coding for antimicrobial effectors, which include the AMPs and lysozymes. After Vip3Aa feeding, unigenes representing all the families of AMPs reported by Pascual et al. [42], were detected up-regulated: cecropins, gloverins (specifically related to the Toll pathway in *S. exigua* [27]), diapausins, moricins, cobatoxins, attacins and lebocins (the last ones reported as not transcriptionally stimulated in *B. mori* midguts after Gram-positive or Gram-negative feeding [66]). In addition, spodoptericins (called defensins in other Lepidoptera) were found up-regulated. Only gallerimycin (an AMP found up-regulated after bacterial challenge in *H. armigera* [67]), although present in the microarray, was not significantly regulated. Moreover, several lysozymes were also up-regulated after Vip3Aa intoxication. 

 Up-regulation of AMPs induced by bacterial infection, bacterial feeding (including *Bacillus* sp), or by Cry toxins, has been previously reported in *S. exigua* [26-28] and in other lepidopteran [16,30,32,48,49,66-68]. In general, the regulated AMPs exhibited an increase in transcription with time following the same pattern previously described for *S. exigua* attacin [28]. The AMP exhibiting the greatest induction was diapausin (up-regulated from 17- to 45-fold depending on the unigene and the time of exposure to Vip3Aa), followed by lebocin and gloverin. The latter has been described as being related to the Bt response in *S. exigua* [27]. Cecropin was the first AMP described in *S. exigua*, and its transcription in the fat body of fifth instar larvae was enhanced after injection of dead bacteria [26]. The different cecropins are grouped into six subfamilies in this Lepidoptera [42] and, although all of them were represented in the microarray, only members of subfamilies B and D were found to be regulated by Vip3Aa feeding.

 Interestingly, we did not observe regulation of the unigenes representing the main immune-related signaling pathways (Toll, IMD, JAK/STAT,p38 MAPK, and JNK MAPK), in an apparent disagreement with the up-regulation observed for AMPs since some of these pathways are responsible for inducing their synthesis. However, the initial activation of these pathways relies mainly on their post-translational modification [19,69]. It would be interesting, in further studies to determine the effect of long-term exposure to Vip3A toxins on the transcriptional regulation of these genes. In fact, Cancino-Rodezno et al. found that MAPK p38 was phosphorylated in *Manduca sexta* larvae after one hour of Cry1Ab exposure, but they only begin to observe an increase in the expression level of MAPKp38 gene after 24 h of the exposure to the toxin [25].

 Vip3Aa feeding also revealed the overexpression of other associated immune-related genes encoding proteins from the Hdd family, such as *Hdd11* and *Hdd23*. For example, *hdd11* was found to be highly induced in midguts of a Cry1Ab-resistant strain of *Diatraea saccharalis* [39], and *Hdd23* was up-regulated in Lepidoptera infected by virus or bacteria [70,71]. Another gene of this family, *Hdd1*, was also up-regulated in midguts of *T. ni* immune-induced by bacterial feeding [16]. The functions of these Hdd proteins remain unclear.

### *Bacillus thuringiensis*-related genes

 The most known insecticidal proteins of Bt are Cry toxins. They have been used, either alone or in combination with Bt, as insecticides for more than 70 years. Despite this long use as biological insecticides, their mode of action has not yet been fully elucidated. To date, it has been demonstrated that Cry proteins are pore forming toxins, and that certain insect midgut proteins are required for them to exert their toxicity [2,10]. The Bt Vip proteins are also pore-forming toxins [12], of which the mode of action is yet unknown. To gain insight into the possible similarities in the intoxication response mechanisms of these two types of Bt toxins, genes encoding proteins linked to Bt or Cry tolerance or resistance, as well as genes involved in the mode of action of Cry and Vip, were sought between the unigenes regulated in response to Vip3Aa feeding.

 To date, two proteins have been implicated in the mode of action of Vip3A in Lepidoptera: an X-tox-like protein [72], and the ribosomal protein S2 [73]. Nine and two unigenes respectively with homology to these protein genes were present in the array but their expression was not altered after Vip3Aa feeding.

 The screened genes related to the Cry toxins mode of action in Lepidoptera included midgut membrane-associated proteins (such as aminopeptidases-N, alkaline phosphatases, cadherin, ABC transporter), intracellular G protein, adenylate cyclase, and protein kinase A [9,74]. Other genes encoding proteins implicated in resistance to Bt or to Cry proteins in Lepidoptera, were also investigated. For example, arylphorin (a storage protein related with immune response elicited by bacterial feeding in *T. ni*, and related with Bt resistance in *S. exigua* [16,33]), members of the REPAT family (correlated with Bt resistance in *S. exigua* [33]), and hexamerin or lipophorin (proteins involved in resistance to Cry1Ac and in tolerance to Cry1Ac and Cry2Ab in *H. armigera* [65,75]). Midgut proteases, which are not only involved in the activation of Bt toxins, but also in many other processes (e.g. digestion), were not included in this screening. Table 4 summarizes the Bt-related unigenes that were found to be regulated.

**Table 4:** Bt related unigenes regulated by Vip3Aa feeding.

**Gene name**	**Unigene**	**Target Name**	**First Hit**	**Fold change 8 h**	**p-value 8 h**	**Fold change 24 h**	**p-value 24 h**
*Aminopeptidase N (APN)*	SE_U10447	SEX-LxN-C6198	gi| 389568606|gb| AFK85027.1| aminopeptidase N-11 [Bombyx mori]	2.92	3.11E-02	4.92	1.51E-03
	SE_U10447	SEX-LxN-C16839	gi |224924544|gb| ACN69218.1 |aminopeptidase N 2 [Mamestra configurata]	2.60	1.12E-01	4.55	6.62E-03
	SE_U07879	SEX-LxN-C17743	gi |327420438|gb| AEA76295.1| aminopeptidase 4A [Mamestra configurata	2.40	1.14E-03	1.88	3.52E-03
	SE_U11572	SEX-LxN-C3476	gi| 345548868|gb| AEO12694.1| aminopeptidase N5 [Ostrinia nubilalis]	2.32	1.93E-03	1.95	4.40E-03
	SE_U07879	SEX-LxN-C9325	gi|30961821|gb|AAP37951.1| midgut aminopeptidase N2 [Helicoverpa armigera]	2.23	2.27E-03	1.87	7.75E-03
*cadherin*	SE_U15248	SEX-LxN-C8315	gi|262527588|gb|ACY69027.1| mutant cadherin [Helicoverpa armigera]	2.00	1.29E-02	2.14	1.71E-02
	SE_U33633	SEX-LxN-C10810	gi|262527588|gb|ACY69027.1| mutant cadherin [Helicoverpa armigera]	1.88	1.03E-02	2.01	2.01E-02
	SE_U33633	SEX-LxN-C14724_sense	gi|262527588|gb|ACY69027.1| mutant cadherin [Helicoverpa armigera]	2.47	2.70E-02	1.65	8.23E-02
*ALP*	SE_U00734	SEX-LxN-C10923	gi|357627201|ref| EHJ76968.1| alkaline phosphatase [Danaus plexippus]	3.42	2.24E-04	2.26	3.34E-04
	SE_U03456	SEX-LxN-C19655	gi|357627201|ref| EHJ76968.1| alkaline phosphatase [Danaus plexippus]	3.07	1.24E-03	2.23	1.18E-02
*ABC transporter*	SE_U26182	SEX-LxN-C9818	gi|327268502|ref| XP_003219036.1| ATP-binding cassette sub-family G member 1-like [Anolis carolinensis]	2.36	2.95E-02	2.99	1.55E-03
	SE_U22657	SEX-LxN-C11260	gi|357605182|ref| EHJ64497.1| putative white family ATP-binding cassette transporter [Danaus plexippus]	-2.85	3.34E-03	-1.87	1.65E-02
	SE_U15221	SEX-LxN-C15152	gi|7381620|gb|AAF61570.1| ATP binding cassette transporter protein [Bombyx mori]	-1.05	4.53E-01	2.16	1.41E-02
	SE_U06473	SEX-LxN-C7701	gi|270209763|gb|ACZ64280.1| ATP-binding cassette sub-family C member 4 [Trichoplusia ni]	-1.27	1.86E-01	-2.01	1.68E-02
	SE_U20037	SEX-LxN-C16152	gi|296427825|ref|ADH16743.1| ABC transporter family C protein ABCC3 [Heliothis subflexa]	-4.07	2.50E-03	-1.45	9.66E-02
*G-protein*	SE_U20617	SEX-LxN-C10504	gi|357624706|ref|EHJ75380.1| G protein-coupled receptor [Danaus plexippus]	2.30	2.34E-04	1.70	1.79E-02
	SE_U13439	SEX-LxN-C15554	gi|ADE43129.1|ref|XP_001355354.2| G-protein coupled receptor [Spodoptera exigua]	1.70	8.46E-03	2.48	2.16E-03
*Adenylate cyclase*	SE_U20781	SEX-LxN-C15236	gi|357623129|ref|EHJ74405.1| adenylate cyclase [Danaus plexippus]	6.65	8.10E-04	3.79	2.62E-03
	SE_U22497	SEX-LxN-C179	gi|91084449|ref|XP_969712.1| similar to adenylate cyclase [Tribolium castaneum]	6.57	1.40E-03	3.68	4.39E-03
	SE_U22441	SEX-LxN-C5034	gi|357624706|ref|EHJ75380.1| G protein-coupled receptor [Danaus plexippus]	1.90	8.20E-02	3.85	5.47E-03
	SE_U08568	SEX-LxN-C5881	gi|357617579|ref| EHJ70872.1 | adenylate cyclase [Danaus plexippus]	1.60	1.46E-01	3.00	4.67E-04
	SE_U11248	SEX-LxN-C9103	gi|389612689|ref|BAM19765.1| adenylate cyclase [Papilio xuthus]	-4.77	8.47E-03	-11.39	1.90E-03
	SE_U14729	SEX-LxN-C1634	gi| 357612641|gb|EHJ68098.1 | adenylate cyclase [Danaus plexippus]	-2.35	3.69E-02	-2.13	6.82E-02
*arylphorin*	SE_U09388	SEX-LxN-C2347	gi|5869989|emb|CAB55605.1| arylphorin subunit [Spodoptera litura]	-7.67	4.10E-02	-7.15	7.22E-02
*apolipophorin*	SE_U36266	SEX-LxN-C1769	gi|347543546|gb|BAK82317.1| apolipophorin precursor protein [Bombyx mori]	-2.36	9.95E-03	-2.21	1.19E-02
	SE_U09762	SEX-LxN-C401	gi|2498144|sp|Q25490.1|APLP_MANSE RecName: Full=Apolipophorins; Contains: RecName: Full=Apolipophorin-2; RecName: Full=Apolipophorin-1	-2.44	9.16E-03	-2.31	1.12E-02
	SE_U22488	SEX-LxN-C7821	gi|2498144|sp|Q25490.1|APLP_MANSE RecName: Full=Apolipophorins; Contains: RecName: Full=Apolipophorin-2; RecName: Full=Apolipophorin-1;	-2.06	1.22E-02	-2.34	1.18E-02
	SE_U09762	SEX-LxN-C13562	gi|347543546|gb|BAK82317.1| apolipophorin precursor protein [Bombyx mori]	-2.40	1.76E-02	-2.34	8.65E-03
	SE_U09762	SEX-LxN-C3447	gi|347543546|gb|BAK82317.1| apolipophorin precursor protein [Bombyx mori]	-2.34	1.67E-02	-2.47	6.17E-03
	SE_U20982	SEX-LxN-C18061	gi|2498144|sp|Q25490.1|APLP_MANSE RecName: Full=Apolipophorins; Contains: RecName: Full=Apolipophorin-2; RecName: Full=Apolipophorin-1	-2.80	1.02E-02	-2.61	5.29E-03
	SE_U40299	SEX-LxN-C3264	gi|300953022|gb|ADK46942.1|apolipophorin-III [Spodoptera exigua]	-2.69	8.69E-03	-1.30	1.79E-01

 After Vip3Aa intoxication, only slight differences in expression (in general overexpression) of some of the genes involved in Bt mode of action were found. This resembles what has been found in studies on whole transcriptional profiles of insect midguts after sublethal Cry intoxication in insects as *C. fumiferana* [30], *S. frugiperda* [31], and the coleopteran *T. molitor* [34], that did not show clear regulation of the putative Cry receptors. Indeed, in the case of *C. fumiferana* some APNs were found down-regulated during early times post-intoxication (5 h) [30].

 Following Vip3Aa intoxication, unigenes homologous to the Cry protein receptor cadherin, were near the cutoff threshold of 2-fold regulation at both time points. The homologues of the ABC transporter (also described as a Cry receptor) showed slight up- and down-regulations, as was described for *B. mori* infected with *B. bombyseptieus* [56]. Unigenes with homology to another family of Cry receptors, the GPI-anchored proteins (APNs and ALPs), were slightly up-regulated. Interestingly, the APN unigenes that were found regulated did not show homology with the five main classes of *S. exigua* midgut APNs described as related with Bt [76]. Regarding the genes not directly related to the binding of the insecticidal protein, there were slight changes in the expression of unigenes homologous to G-protein and adenylatecyclase after Vip3Aa feeding. The exceptions were some down-regulated unigenes homologous to adenylate cyclase. The lack of significant changes in the transcription levels of all the Bt-mode of action related genes may suggest that they are not involved in the Vip mode of action. Alternatively, it may also indicate that, if the mode of action of Cry and Vip toxins share biochemical processes, the mechanisms of defense against Vip toxins would not rely on transcriptional regulation of the members involved in them.

 Apolipophorin was found slightly down-regulated during the entire course of Vip3Aa intoxication. This contrasts with studies performed in immune-induced lepidopterans (such as *T. ni* fed with bacteria [16]), or with coleopterans (such as *T. molitor* fed with Cry3Aa [34] or *T. castaneum* fed with Cry3Aa or Cry23Aa/Cry37Aa [37]), where *apolipophorin III* was up-regulated. Indeed, Cry1Ab-resistant *D. saccharalis* [39] showed constitutive up-regulation of an apolipophorin precursor. Typically, lipophorins I and II are considered insect hemolymph proteins involved in lipid transport, but lipophorin III has been also implied in defense mechanisms by clotting [21,61]. In *H. armigera*, it has been shown that, in general, lipophorins can bind to Cry1Ac and Cry2Ab monomers and sequester the toxins [75]. The nature of the down-regulation of apolipophorins detected after Vip3Aa feeding is likely due to their role as lipid transporters, and their low levels during feeding could be a consequence of the general reduction of metabolic processes caused by feeding cessation.

Arylphorin is a hexamerin related to the immune response because of its mitogen activity, which is associated with cell proliferation and the replacement of damaged cells [77]. Arylphorin was found to be up-regulated in a Bt-resistant *S. exigua* colony [33], in *S. exigua* after Bt intoxication [33], and in a Cry1Ab-resistant *D. saccharalis* strain [39]. In our experiments, *arylphorin* was down-regulated in Vip3Aa-treated larvae. The different mode of action of Cry and Vip toxins could be the reason for these different observations, or, alternatively, it could be that the regulation observed in *arylphorin* was independent of Bt, Cry or Vip intoxication.

REPATs are midgut infection-response glycoproteins that were first discovered in *S. exigua*, up-regulated after treatment with different Bt toxins or with baculovirus [29], and that were overexpressed in an insect colony resistant to Bt formulations [33]. Recently, up to 46 members of this family have been reported in *S. exigua*, and homologous sequences have been found in other species [54]. After Vip3Aa feeding, a broad response of *repat* genes was detected. Unigenes with homologies to 29 different *repat* genes were found regulated, which pointed to a strong involvement of these genes in the midgut response to Vip3Aa (Table 5). In general, *repat* unigenes were overexpressed, exhibiting about the same level of up-regulation at 8 and 24 hours. A clearly different behavior was exhibited by *repat42, repat43*, *repat46* and another *repat* unigene with low homology to *repat14* (e-value = 0.01, which indicates it is likely a new member of the family), which were down-regulated.

**Table 5:** Unigenes with homology to *repat* genes regulated by Vip3Aa feeding.

**Unigene**	**Target**	**Blastx First Hit**	**e value**	**Fold change 8 h**	**p-value 8 h**	**Fold change 24 h**	**p-value 24 h**
SE_U11312	SEX-LxN-C3620_sense	gi|383931963|gb|AFH57143.1| REPAT23 [Spodoptera exigua]	3E-92	18.6	1.57E-03	163.03	1.72E-04
SE_U09334	SEX-LxN-C111	gi|209868384|gb|ACI90727.1|REPAT2 [Spodoptera exigua]	3E-87	16.38	1.58E-03	36.28	2.12E-04
SE_U20685	SEX-LxN-C17485	gi|209868384|gb|ACI90727.1| REPAT2 [Spodoptera exigua]	3E-46	14.29	1.26E-03	22.21	6.80E-04
SE_U20782	SEX-LxN-C2522	gi|383931949|gb|AFH57136.1| REPAT16 [Spodoptera exigua]	4E-82	12.74	8.52E-04	20.02	5.00E-81
SE_U40281	SEX-LxN-C2017	gi|383931939|gb|AFH57131.1| REPAT11 [Spodoptera exigua]	9E-92	6.81	2.02E-03	9.68	4.40E-04
SE_U10302	SEX-LxN-C449	gi|383931963|gb|AFH57143.1| REPAT23 [Spodoptera exigua]	6E-97	4.6	4.69E-04	9.45	7.56E-05
SE_U10977	SEX-LxN-C1968	gi|383931943|gb|AFH57133.1| REPAT13 [Spodoptera exigua]	4E-43	11.23	3.26E-04	8.79	6.78E-05
SE_U19481	SEX-LxN-C16997	gi|209868382| gb|ACI90726.1| REPAT1 [Spodoptera exigua]	1E-80	40.41	2.86E-03	8.39	5.51E-02
SE_U07973	SEX-LxN-C19798	gi|134148365| gb| ABO64233.1| Repat3 [Spodoptera exigua]	6E-33	5.99	2.32E-03	8.07	8.87E-04
SE_U28551	SEX-LxN-C19326	gi|383931942|gb|JQ619195.1| REPAT13 [Spodoptera exigua]	5E-25	12.10	3.45E-04	6.24	2.69E-04
SE_U50962	SEX-LxN-C1906	gi|383931935|gb| AFH57129.1| REPAT9 [Spodoptera exigua]	5E-48	6.24	9.85E-03	6.02	1.04E-02
SE_U14074	SEX-LxN-C20119	gi|239809560|gb|ACS26247.1| repat6 [Spodoptera exigua]	3E-83	4.88	3.74E-04	5.99	1.74E-05
SE_U09088	SEX-LxN-C1874	gi|383931935|gb|AFH57129.1| REPAT9 [Spodoptera exigua]	7E-21	4.67	3.20E-03	5.78	1.73E-03
SE_U38915	SEX-LxN-C1910	gi|239809560|gb|ACS26247.1| repat6 [Spodoptera exigua]	2E-89	4.09	1.86E-04	5.28	1.75E-05
SE_U14596	SEX-LxN-C19227	gi|239809560|gb|ACS26247.1| repat6 [Spodoptera exigua]	3E-89	4.21	3.30E-04	5.24	1.62E-05
SE_U20583	SEX-LxN-C693	gi|134148367|gb|ABO64234.1| Repat4 [Spodoptera exigua]	2E-26	4.10	4.43E-03	4.78	2.63E-03
SE_U21875	SEX-LxN-C19838	gi|383931935|gb| AFH57129.1| REPAT9 [Spodoptera exigua]	6E-95	4.88	1.23E-02	4.70	1.33E-02
SE_U14129	SEX-LxN-C2973	gi|383931965|gb|AFH57144.1|REPAT24 [Spodoptera exigua]	2E-105	3.36	4.37E-03	4.35	1.52E-03
SE_U21293	SEX-LxN-C4254	gi|209868382|gb|ACI90726.1| REPAT1 [Spodoptera exigua]	5E-80	20.33	3.59E-03	4.14	1.25E-03
SE_U06640	SEX-LxN-C508	gi|383931947|gb|AFH57135.1| REPAT15 [Spodoptera exigua]	3E-107	4.11	1.28E-03	3.74	1.57E-03
SE_U10737	SEX-LxN-C1994	gi|383931953|gb|AFH57138.1 | REPAT18 [Spodoptera exigua]	4E-90	3.15	4.68E-03	3.58	2.00E-03
SE_U22330	SEX-LxN-C2171	gi|383931937|gb|AFH57130.1| REPAT10 [Spodoptera exigua]	6E-92	3.25	2.39E-02	3.38	2.15E-02
SE_U18260	SEX-LxN-C20125	gi|383931959|gb|AFH57141.1|REPAT21 [Spodoptera exigua]	8E-73	4.85	6.61E-04	3.31	1.77E-03
SE_U42150	SEX-LxN-C1416	gi|383931955|gb|AFH57139.1| REPAT19 [Spodoptera exigua]	2E-88	3.87	8.41E-05	3.29	1.48E-05
SE_U13940	SEX-LxN-C13269_sense	gi|383931955|gb|AFH57139.1| REPAT19 [Spodoptera exigua]	3E-33	3.99	8.76E-05	3.28	5.10E-05
SE_U36203	SEX-LxN-C1775	gi|239809558|gb|ACS26246.1| repat5 [Spodoptera exigua]	3E-98	2.69	1.81E-03	3.27	1.04E-03
SE_U04530	SEX-LxN-C2214	gi|383931959|gb|AFH57141.1| REPAT21 [Spodoptera exigua]	7E-74	4.46	7.83E-04	3.20	1.86E-03
SE_U31820	SEX-LxN-C3173	gi|340541635|gb|AEK50323.1| REPAT8 [Spodoptera exigua]	6E-91	2.97	2.46E-02	3.06	2.29E-02
SE_U19524	SEX-LxN-C1873	gi|383931941|gb|AFH57132.1| REPAT12 [Spodoptera exigua]	4E-76	2.46	1.02E-02	2.58	7.99E-03
SE_U12488	SEX-LxN-C20059	gi|383931957|gb|AFH57140.1| REPAT20 [Spodoptera exigua]	5E-88	4.13	3.36E-03	2.41	2.23E-02
SE_U27341	SEX-LxN-C19198	gi|383932007|gb|AFH57165.1|REPAT45 [Spodoptera exigua]	3E-88	2.29	1.94E-02	2.36	1.74E-02
SE_U09840	SEX-LxN-C1750	gi|383931967|gb|AFH57145.1|REPAT25 [Spodoptera exigua]	4E-104	2.62	5.39E-03	2.35	3.93E-03
SE_U11591	SEX-LxN-C2036	gi|383931945|gb|AFH57134.1|REPAT14 [Spodoptera exigua]	1E-115	3.26	2.99E-03	2.13	6.92E-03
SE_U10393	SEX-LxN-C3382	gi|383931969|gb|AFH57146.1|REPAT26 [Spodoptera exigua]	1E-96	7.73	2.08E-03	2.04	4.46E-02
SE_U35420	SEX-LxN-C67	gi|383931983|gb|AFH57153.1|REPAT33 [Spodoptera exigua]	6E-71	3.01	4.42E-05	1.95	3.93E-04
SE_U11058	SEX-LxN-C1819	gi|383931995|gb|AFH57159.1|REPAT39 [Spodoptera exigua]	3E-73	-1.28	9.74E-02	2.01	7.77E-03
SE_U33185	SEX-LxN-C3255	gi|383931983|gb|AFH57153.1|REPAT33 [Spodoptera exigua]	2E-70	2.47	2.77E-05	1.79	5.62E-04
SE_U21578	SEX-LxN-C1765	gi|383932009|gb|AFH57166.1|REPAT46 [Spodoptera exigua]	4E-83	-1.81	8.51E-02	-2.91	3.15E-02
SE_U27212	SEX-LxN-C11057_anti	gi|383931945|gb|AFH57134.1| REPAT14 [Spodoptera exigua]	0.01	-6.15	1.63E-03	-3.86	5.61E-03
SE_U00804	SEX-LxN-C4755	gi|383932001|gb|AFH57162.1|REPAT42 [Spodoptera exigua]	6E-53	-2.72	2.74E-02	-8.86	2.70E-03
SE_U10377	SEX-LxN-C4998	gi|383932003|gb|AFH57163.1|REPAT43 [Spodoptera exigua]	4E-60	-4.35	3.16E-03	-40.28	1.36E-04

Almost all *repat* members that were significantly regulated belonged to REPAT class α (groups I and II), and only five of them belonged to REPAT class ß (to groups III, IV, and VI) [54]. The general up-regulation of the *repat* unigenes from class α together with *repat45*, and the down-regulation of class ß *repat* members 42 and 43, coincide with the transcriptional profiles reported in *S. exigua* treated with Cry1C by Navarro-Cerrillo et al. [54]. Although the role of REPAT proteins remains unclear, the large number of *repat* members regulated, their homology to transcriptional activators in other species of Lepidoptera, and their ability to form heterodimers and translocate into the nucleus [78] seems to point to a possible role in the transcriptional activation of several sets of genes in response to physiological changes in the midgut produced by Vip3A or Cry1C intoxication.

In summary, in this work, the overall transcriptional response of the midgut of a lepidopteran such as *S. exigua* exposed to the toxic action of Vip3Aa has been described for the first time. A comprehensive response characterized by the overexpression of immune-related unigenes and *repat* family unigenes, was detected together with other singular regulations (e.g. up-regulation of hormone-binding protein unigenes or down-regulation of serin proteases and chitin deacetylases). The data reported here may contribute to a better understanding of the interaction of the insect midgut with the Vip3Aa toxin, helping to unravel the processes underlying Vip toxicity. This information may allow for the design of more effective pest-management strategies using this toxin (alone or in combination with other insecticidal agents), e.g. pointing to larval genes involved in resistance mechanisms, or to targets for RNAi mediated gene disruption. In short, it may provide new tools for crop protection.

## Supporting Information

Figure S1 **Growth inhibition dose-response curve of *S.exigua* newly moulted L4 larvae challenged with Vip3Aa.** Growth inhibition values were calculated following Herrero et al. [79]. Four biological replicates of the experiment (using 8 larvae per dose) were performed.
(TIFF)

Figure S2 **Confirmation of microarray results by qRT-PCR.** Graphs show the fold-change values obtained by microarray (solid lines) versus expression ratio values obtained by qRT-PCR (dotted lines) for each validated ESTs.
(TIF)

Figure S3 **Sequence alignment of the S. *exigua* hypothetical protein REVIP (GeneBank Acc. No. KF601929), and the *H. armigera* (GeneBank Acc. No. BU038696) and *B. mori* (Silkworm Genome database BGIBMGA010981-TA) homologues, using ClustalX2 [80].**
(TIF)

Table S1 **Primers used for qRT-PCR amplifications.**
(DOCX)

Table S2 **Temporal expression clusters of regulated unigenes generated by Babelomics software.**
(XLSX)

Table S3 ***S. exigua* unigenes with homology to sequences in public databases, regulated by Vip3Aa feeding.** The data are distributed into three sections: one for unigenes regulated at 8 h and 24 h, a second section for unigenes regulated only at 8 h, and a third section for unigenes regulated only at 24 h.
(XLSX)

Table S4 **Functional annotation clusters of genes regulated by Vip3Aa feeding, generated by DAVID software, with enrichment score values lower than 0.5.**
(DOCX)
