# Supplementary figures and images for: Comprehensive Analysis of Gene Expression Profiles of the Beet Armyworm Spodoptera exigua Larvae Challenged with Bacillus thuringiensis Vip3Aa Toxin
Source: PLoS One. 2013 Dec 2;8(12):e81927. doi: 10.1371/journal.pone.0081927 (PMC3846680; doi:10.1371/journal.pone.0081927)

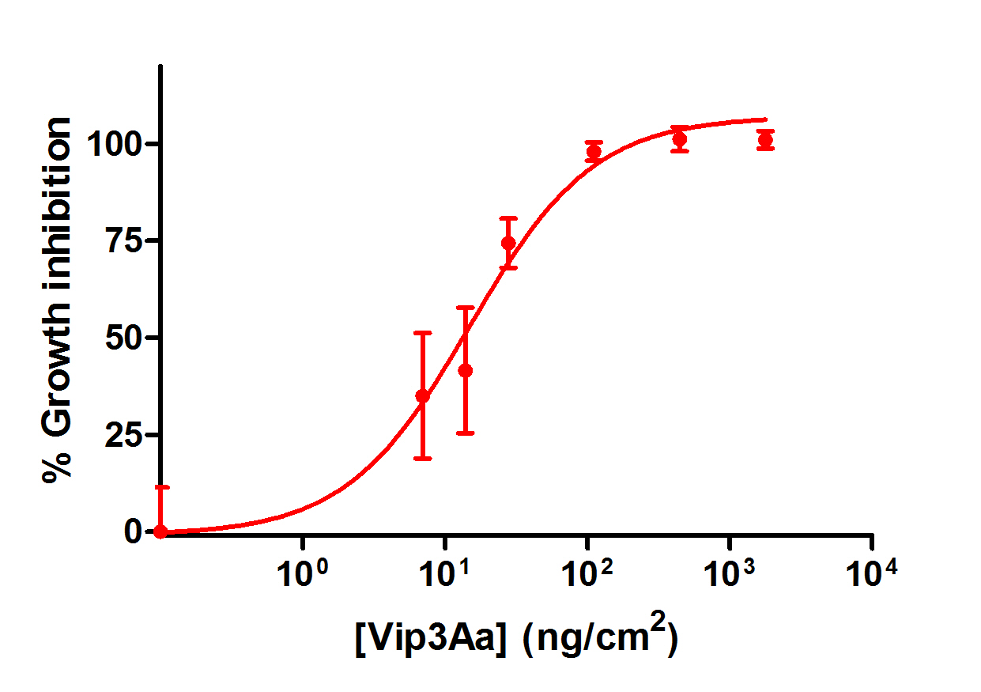

Supplement: Figure S1 — Growth inhibition dose-response curve of S.exigua newly moulted L4 larvae challenged with Vip3Aa. Growth inhibition values were calculated following Herrero et al. [79]. Four biological replicates of the experiment (using 8 larvae per dose) were performed. (TIFF) [file pone.0081927.s001.tiff]

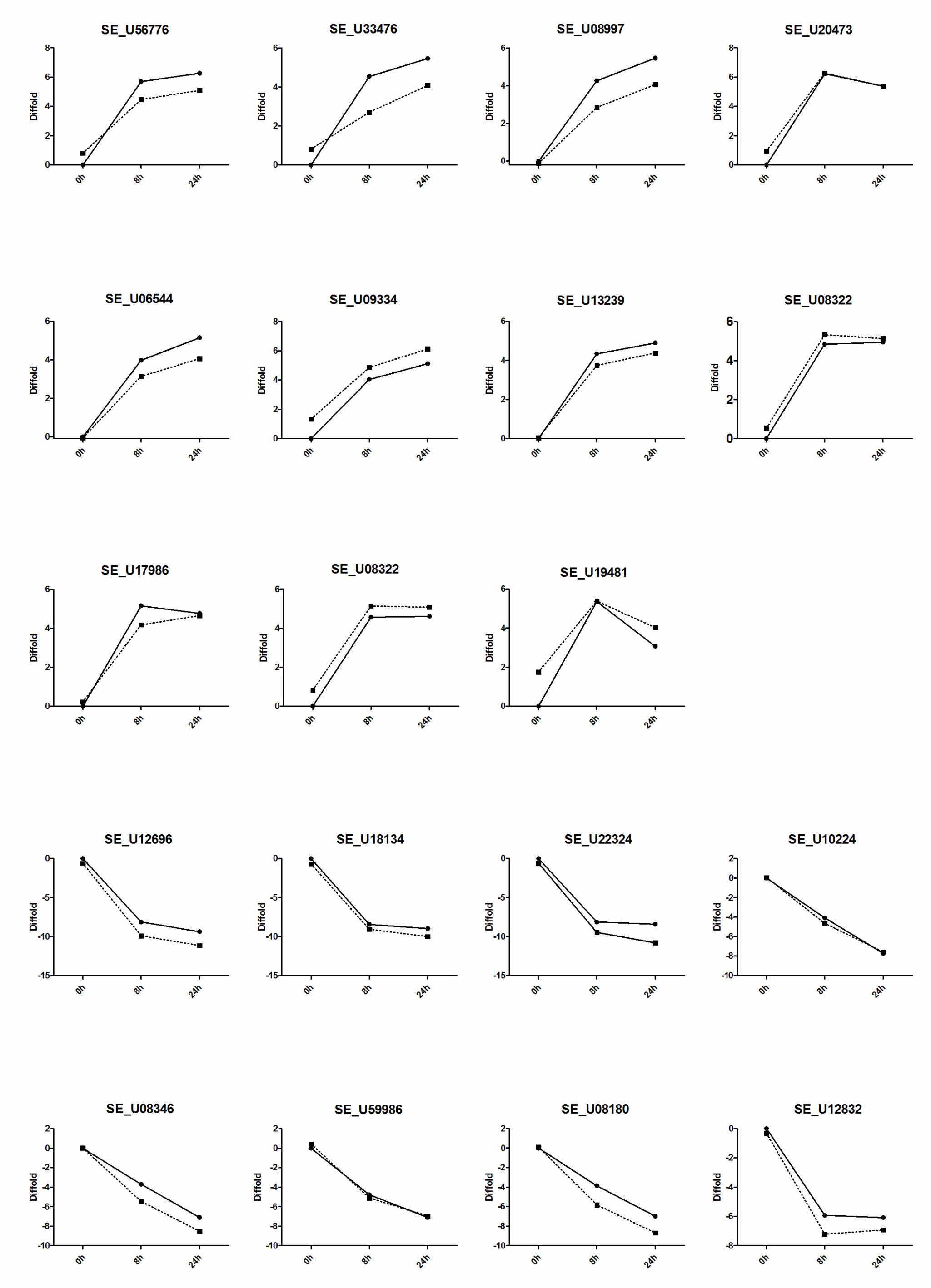

Supplement: Figure S2 — Confirmation of microarray results by qRT-PCR. Graphs show the fold-change values obtained by microarray (solid lines) versus expression ratio values obtained by qRT-PCR (dotted lines) for each validated ESTs. (TIF) [file pone.0081927.s002.tif]

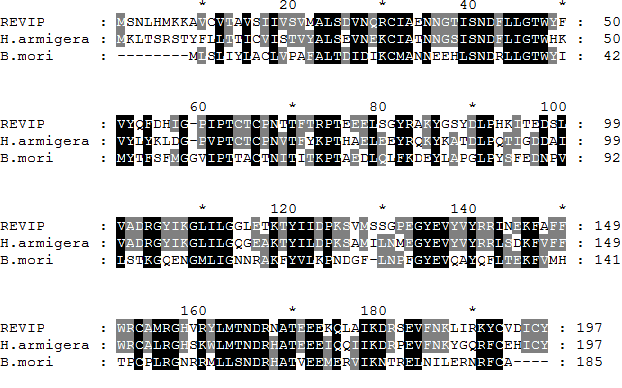

Supplement: Figure S3 — Sequence alignment of the S. exigua hypothetical protein REVIP (GeneBank Acc. No. KF601929), and the H. armigera (GeneBank Acc. No. BU038696) and B. mori (Silkworm Genome database BGIBMGA010981-TA) homologues, using ClustalX2 [80]. (TIF) [file pone.0081927.s003.tif]
