# Supplementary material for: Comprehensive Analysis of Gene Expression Profiles of the Beet Armyworm Spodoptera exigua Larvae Challenged with Bacillus thuringiensis Vip3Aa Toxin
Source: PLoS One. 2013 Dec 2;8(12):e81927. doi: 10.1371/journal.pone.0081927 (PMC3846680; doi:10.1371/journal.pone.0081927)
Supplement: Table S1 — Primers used for qRT-PCR amplifications. (DOCX) [file pone.0081927.s004.docx]

**Table S1. Primers used for the qRT-PCR amplifications.**

| **Target gene** | **Forward (5’ → 3’)** | **Reverse (5’ → 3’)** |
| --- | --- | --- |
| *ATP synthase* | GTTGCTGGTCTGGTGGGATT | AGGCCTCAGACACCATTGAAA |
| SE_U17986 | CGAGTGCACCATGAACACCT | ATGACGGCGAGGAAAGAGAG |
| SE_U33476 | CAGTACAATGGCCGCTCTCAA | AAGGCAATGAGGAGCAGCAC |
| SE_U 08997 | CTCCCCGAAGCTGAGACCT | TGGTCTCCGGCTTTATTGGA |
| SE_U20473 | AGACATCCAGCCATCTTAACACC | GGGCGACGTGGACTTCTATC |
| SE_U06544 | TCAATTTCCAATAAAGCCGGA | TCTCGTGCTCAGCAATGTGC |
| SE_U09334 | CAGTCGCCGGCCAAATAC | CGGGCTCGGCTTTATAGACC |
| SE_U13239 | TTGGGCATCAAGTCGCTAGA | GTCCCCCTTGATCTCGTCAA |
| SE_U08322 | GCCGCTAAGAATGCAGCTAAA | TGATGCCCGTGGAAGCTT |
| SE_U17986 | AATTGGCGTGATGTTATGCG | GATGGCGTGAACAATTGGTG |
| SE_U20473 | CGGCCAAGAATTAGTTTCCAAA | AGACCGGGTACTCTGGCGTA |
| SE_U19481 | TAGGTACGAGGGTTGGGTGC | GCTGGCGCGAAATCGTT |
| SE_U12696 | GGTCCAATTCCAACATGCACT | TGTAGGTCTTGTGAACGTGGTGT |
| SE_U18134 | AAGAACAATGAGCAACTCGCAC | ACAGCAGTTACACAAACTGCCTTC |
| SE_U22324 | AACGACAGGAATGCCACTGAG | TCGGTCTTTAATGGCAAGCTG |
| SE_U10224 | CGAAGGGAATGTTTGCGAAG | AGTTCGCTGACCAGAGAGTGC |
| SE_U08346 | AGGTCATCTCCAGCTACGACG | CGTTGCACGATTCAAATTCG |
| SE_U59986 | GCCATTGCCTTACCTTCTGG | GCTTCCAACAAAGTTCTCGTTGA |
| SE_U08180 | ATTCGCCCGACCTCTTCAAT | TGTTTAGGATGAACTGGAACCATAAC |
| SE_U12832 | ACTGGTGCAGTTCCGAGCAT | AGCCCCAATACTGTGTCCCA |
