# Supplementary material for: Comprehensive Analysis of Gene Expression Profiles of the Beet Armyworm Spodoptera exigua Larvae Challenged with Bacillus thuringiensis Vip3Aa Toxin
Source: PLoS One. 2013 Dec 2;8(12):e81927. doi: 10.1371/journal.pone.0081927 (PMC3846680; doi:10.1371/journal.pone.0081927)
Supplement: Table S4 — Functional annotation clusters of genes regulated by Vip3Aa feeding, generated by DAVID software, with enrichment score values lower than 0.5. (DOCX) [file pone.0081927.s007.docx]

**Table S4. Functional annotation clusters of genes that are regulated by Vip3Aa feeding, with enrichment scores lower than 0.5.**

| **Annotation Cluster** | **Term** | **Nr of genes in this cluster** | **% of genes in this cluster** | **p-value** |
| --- | --- | --- | --- | --- |
| Cluster 8 | Enrichment Score: 0.39 |  |  |  |
|  | GO:0016021~integral to membrane | 9 | 0.94 | 2.51E-01 |
|  | GO:0031224~intrinsic to membrane | 9 | 0.94 | 2.65E-01 |
|  | membrane | 7 | 0.73 | 4.29E-01 |
|  | transmembrane | 5 | 0.52 | 9.85E-01 |
| Cluster 9 | Enrichment Score: 0.36 |  |  |  |
|  | GO:0034613~cellular protein localization | 3 | 0.31 | 4.00E-01 |
|  | GO:0006886~intracellular protein transport | 3 | 0.31 | 4.00E-01 |
|  | GO:0070727~cellular macromolecule localization | 3 | 0.31 | 4.00E-01 |
|  | GO:0015031~protein transport | 4 | 0.42 | 4.45E-01 |
|  | GO:0045184~establishment of protein localization | 4 | 0.42 | 4.45E-01 |
|  | GO:0008104~protein localization | 4 | 0.42 | 4.45E-01 |
|  | GO:0046907~intracellular transport | 3 | 0.31 | 5.00E-01 |
| Cluster 10 | Enrichment Score: 0.34 |  |  |  |
|  | GO:0005509~calcium ion binding | 4 | 0.42 | 3.13E-01 |
|  | IPR002048:Calcium-binding EF-hand | 3 | 0.31 | 4.29E-01 |
|  | SM00054:EFh | 3 | 0.31 | 4.50E-01 |
|  | IPR018249:EF-HAND 2 | 3 | 0.31 | 4.63E-01 |
|  | IPR011992:EF-Hand type | 3 | 0.31 | 4.96E-01 |
|  | IPR018247:EF-HAND 1 | 3 | 0.31 | 5.27E-01 |
|  | calcium | 3 | 0.31 | 5.57E-01 |
| Cluster 11 | Enrichment Score: 0.32 |  |  |  |
|  | GO:0044271~nitrogen compound biosynthetic process | 5 | 0.52 | 4.04E-01 |
|  | GO:0009165~nucleotide biosynthetic process | 4 | 0.42 | 4.25E-01 |
|  | GO:0034404~nucleobase, nucleoside and nucleotide biosynthetic process | 4 | 0.42 | 4.45E-01 |
|  | GO:0034654~nucleobase, nucleoside, nucleotide and nucleic acid biosynthetic process | 4 | 0.42 | 4.45E-01 |
|  | GO:0009260~ribonucleotide biosynthetic process | 3 | 0.31 | 6.09E-01 |
|  | GO:0009259~ribonucleotide metabolic process | 3 | 0.31 | 6.09E-01 |
| Cluster 12 | Enrichment Score: 0.27 |  |  |  |
|  | GO:0030414~peptidase inhibitor activity | 5 | 0.52 | 3.87E-01 |
|  | GO:0004857~enzyme inhibitor activity | 5 | 0.52 | 4.43E-01 |
|  | GO:0004866~endopeptidase inhibitor activity | 4 | 0.42 | 5.87E-01 |
|  | GO:0004867~serine-type endopeptidase inhibitor activity | 4 | 0.42 | 5.87E-01 |
|  | IPR000215:Protease inhibitor I4, serpin | 4 | 0.42 | 6.06E-01 |
|  | SM00093:SERPIN | 4 | 0.42 | 6.37E-01 |
| Cluster 13 | Enrichment Score: 0.20 |  |  |  |
|  | IPR010987:Glutathione S-transferase, C-terminal-like | 3 | 0.31 | 5.27E-01 |
|  | IPR004045:Glutathione S-transferase, N-terminal | 3 | 0.31 | 6.13E-01 |
|  | IPR017933:Glutathione S-transferase/chloride channel, C-terminal | 3 | 0.31 | 6.39E-01 |
|  | IPR012335:Thioredoxin fold | 3 | 0.31 | 7.67E-01 |
| Cluster 14 | Enrichment Score: 0.19 |  |  |  |
|  | GO:0022890~inorganic cation transmembrane transporter activity | 4 | 0.42 | 4.89E-01 |
|  | GO:0006812~cation transport | 3 | 0.31 | 6.66E-01 |
|  | GO:0006811~ion transport | 3 | 0.31 | 8.49E-01 |
| Cluster 15 | Enrichment Score: 0.17 |  |  |  |
|  | GO:0016310~phosphorylation | 5 | 0.52 | 5.66E-01 |
|  | GO:0006793~phosphorus metabolic process | 5 | 0.52 | 6.38E-01 |
|  | GO:0006796~phosphate metabolic process | 5 | 0.52 | 6.38E-01 |
|  | GO:0032559~adenyl ribonucleotide binding | 6 | 0.63 | 8.02E-01 |
|  | GO:0005524~ATP binding | 6 | 0.63 | 8.02E-01 |
| Cluster 16 | Enrichment Score: 0.15 |  |  |  |
|  | GO:0005856~cytoskeleton | 3 | 0.31 | 3.82E-01 |
|  | GO:0043228~non-membrane-bounded organelle | 4 | 0.42 | 9.80E-01 |
|  | GO:0043232~intracellular non-membrane-bounded organelle | 4 | 0.42 | 9.80E-01 |
| Cluster 17 | Enrichment Score: 0.12 |  |  |  |
|  | Initiation factor | 3 | 0.31 | 6.86E-01 |
|  | GO:0003743~translation initiation factor activity | 3 | 0.31 | 7.17E-01 |
|  | GO:0008135~translation factor activity, nucleic acid binding | 3 | 0.31 | 8.67E-01 |
| Cluster 18 | Enrichment Score: 0.09 |  |  |  |
|  | GO:0006355~regulation of transcription, DNA-dependent | 4 | 0.42 | 7.12E-01 |
|  | GO:0051252~regulation of RNA metabolic process | 4 | 0.42 | 7.12E-01 |
|  | GO:0043565~sequence-specific DNA binding | 3 | 0.31 | 7.78E-01 |
|  | DNA-binding | 4 | 0.42 | 7.99E-01 |
|  | GO:0045449~regulation of transcription | 4 | 0.42 | 8.31E-01 |
|  | GO:0003700~transcription factor activity | 3 | 0.31 | 8.39E-01 |
|  | GO:0030528~transcription regulator activity | 4 | 0.42 | 8.52E-01 |
|  | GO:0003677~DNA binding | 5 | 0.52 | 9.04E-01 |
|  | nucleus | 4 | 0.42 | 9.39E-01 |
| Cluster 19 | Enrichment Score: 0.08 |  |  |  |
|  | GO:0001883~purine nucleoside binding | 7 | 0.73 | 7.70E-01 |
|  | GO:0001882~nucleoside binding | 7 | 0.73 | 7.70E-01 |
|  | GO:0030554~adenyl nucleotide binding | 7 | 0.73 | 7.70E-01 |
|  | GO:0017076~purine nucleotide binding | 10 | 1.04 | 7.84E-01 |
|  | GO:0032559~adenyl ribonucleotide binding | 6 | 0.63 | 8.02E-01 |
|  | GO:0005524~ATP binding | 6 | 0.63 | 8.02E-01 |
|  | GO:0032553~ribonucleotide binding | 9 | 0.94 | 8.07E-01 |
|  | GO:0032555~purine ribonucleotide binding | 9 | 0.94 | 8.07E-01 |
|  | ATP-binding | 4 | 0.42 | 9.11E-01 |
|  | GO:0000166~nucleotide binding | 12 | 1.25 | 9.33E-01 |
|  | nucleotide-binding | 5 | 0.52 | 9.79E-01 |
| Cluster 20 | Enrichment Score: 0.07 |  |  |  |
|  | GO:0070011~peptidase activity, acting on L-amino acid peptides | 5 | 0.52 | 7.71E-01 |
|  | GO:0008233~peptidase activity | 5 | 0.52 | 8.28E-01 |
|  | GO:0006508~proteolysis | 4 | 0.42 | 9.25E-01 |
|  | GO:0004175~endopeptidase activity | 3 | 0.31 | 9.28E-01 |
| Cluster 21 | Enrichment Score: 0.06 |  |  |  |
|  | GO:0019001~guanyl nucleotide binding | 3 | 0.31 | 8.76E-01 |
|  | GO:0032561~guanyl ribonucleotide binding | 3 | 0.31 | 8.76E-01 |
|  | GO:0005525~GTP binding | 3 | 0.31 | 8.76E-01 |
